# Convergent selective signaling impairment exposes the pathogenicity of latrophilin-3 missense variants linked to inheritable ADHD susceptibility

**DOI:** 10.1038/s41380-022-01537-3

**Published:** 2022-04-07

**Authors:** Ana L. Moreno-Salinas, Brian J. Holleran, Estefania Y. Ojeda-Muñiz, Kerlys G. Correoso-Braña, Sheila Ribalta-Mena, José-Carlos Ovando-Zambrano, Richard Leduc, Antony A. Boucard

**Affiliations:** 1 Department of Cell Biology, Centro de Investigación y de Estudios Avanzados del Instituto Politécnico Nacional (Cinvestav-IPN), México City, México; 2 Department of Pharmacology-Physiology, Faculty of Medicine and Health Sciences, Université de Sherbrooke, Sherbrooke, Canada; 3 Department of Physiology, Biophysics and Neurosciences, Centro de Investigación y de Estudios Avanzados del Instituto Politécnico Nacional (Cinvestav-IPN), México City, México

**Keywords:** Cell biology, Neuroscience

## Abstract

Latrophilin-3 (Lphn3; also known as ADGRL3) is a member of the adhesion G Protein Coupled Receptor subfamily, which participates in the stabilization and maintenance of neuronal networks by mediating intercellular adhesion through heterophilic interactions with transmembrane ligands. Polymorphisms modifying the Lphn3 gene are associated with attention-deficit/hyperactivity disorder (ADHD) in children and its persistence into adulthood. How these genetic alterations affect receptor function remains unknown. Here, we conducted the functional validation of distinct ADHD-related Lphn3 variants bearing mutations in the receptor’s adhesion motif-containing extracellular region. We found that all variants tested disrupted the ability of Lphn3 to stabilize intercellular adhesion in a manner that was distinct between ligands classes, but which did not depend on ligand-receptor interaction parameters, thus pointing to altered intrinsic receptor signaling properties. Using G protein signaling biosensors, we determined that Lphn3 couples to Gαi1, Gαi2, Gαs, Gαq, and Gα13. However, all ADHD-related receptor variants consistently lacked intrinsic as well as ligand-dependent Gα13 coupling efficiency while maintaining unaltered coupling to Gαi, Gαs, and Gαq. Consistent with these alterations, actin remodeling functions as well as actin-relevant RhoA signaling normally displayed by the constitutively active Lphn3 receptor were impeded by select receptor variants, thus supporting additional signaling defects. Taken together, our data point to Gα13 selective signaling impairments as representing a disease-relevant pathogenicity pathway that can be inherited through Lphn3 gene polymorphisms. This study highlights the intricate interplay between Lphn3 GPCR functions and the actin cytoskeleton in modulating neurodevelopmental cues related to ADHD etiology.

## Introduction

Attention deficit hyperactivity disorder (ADHD) is a complex neurodevelopmental condition with a strong genetic component that is symptomatically defined by a set of behavioral phenotypes such as impulsivity, inattention, and hyperactivity [1]. Brain imaging of ADHD patients displays ultrastructural abnormalities modifying various interconnected key brain regions related to attention and motor planning such as components of the cortico-striato-thalamo-cortical loop or the limbic system, thus pointing to an underlying defect in neuronal connections in specific brain nuclei and more importantly suggesting a dysregulation of as yet unknown molecular determinants of cellular function [2–8]. Establishing accurate and functional neuronal networks during development relies on adhesion molecules acting both as guidance cues to generate migration patterns and as target selection factors to anchor intercellular contacts that will undergo further maturation [9]. Such a process is suspected to be deficient in ADHD affected brains but the precise cellular and molecular defects leading to this condition are currently unknown. Attempts to identify such factors based on genetic linkage studies conducted on ADHD cohorts worldwide led to the identification of genes encoding reward pathway-related elements linked to dopamine signaling as well as adhesion molecules for which the role in ADHD etiology remains elusive [10].

Latrophilin-3 (Lphn3; also known as ADGRL3), a member of the adhesion G protein-coupled receptor (aGPCR) subfamily, emerged as one potential candidate protein encoded within the ADHD susceptibility haplotype contained in a 325 kb minimal critical region in human genomes [10]. Latrophilins comprise 3 isoforms in mammals (Lphn1, Lphn2, and Lphn3), all of which are mainly expressed in brain tissues. Consistent with their adhesion function in neurons, latrophilins have been described as important synapse organizers, a role that they fulfill by establishing heterophilic contacts with adhesion molecules from distinct families such as teneurins, fibronectin leucine-rich transmembrane protein (FLRT) and neurexins [11–15]. Their ligand-binding activity has important functional implications for neuronal networks in that it determines the degree of stability and maintenance of excitatory synapses in the hippocampus [16]. Olfactomedin and lectin domains, located within their N-terminal extracellular region, recapitulate most of the adhesive functions that are elicited by these receptors while the C-terminal region adopts a seven transmembrane tertiary structure typical of GPCRs. An intermediate region known as the GPCR Autoproteolytic INducing (GAIN) domain in which nests an intramolecular cleavage site separating both N-terminal and C-terminal regions generates distinct protein fragments, NTF and CTF respectively. The GAIN domain simultaneously establishes non-covalent bonds in order to preserve the bipartite complex between the two proteolytically-cleaved fragments [17]. A complex interplay involving both NTF and CTF regions converts adhesive contacts into intracellular signaling cascades mediated by G protein modulation of second messengers or other cellular effector molecules [18–21]. A sequence located immediately C-terminal to the autoproteolytic cleavage site is thought to act as a cryptic CTF ligand embedded within the NTF which, once uncovered, is responsible for inducing receptor-mediated signaling [22, 23]. Such signaling has been described both in presence and absence of known ligands, thus suggesting that latrophilins possess the intrinsic ability to adopt active conformations exhibiting constitutive activity toward intracellular effectors [18, 22, 24]. Constitutive signaling implicating Lphn3 includes the regulation of actin dynamics and activation of subsets of G proteins [18, 22]. While the signaling properties of latrophilins involve coupling to G proteins and actin remodeling, their full signaling profile remains incomplete.

ADHD genetic linkage analysis and further sequencing of Lphn3 gene revealed defects targeting a region which mostly comprises the exons encoding the NTF and interconnecting introns [25]. These genetic alterations are represented by synonymous as well as non-synonymous mutations. Modifications which occur in untranslated upstream regions unveiled potential enhancer-modifying properties as their presence affects transcriptional activity leading to a decrease of Lphn3 expression thus generating a loss-of-function phenotype [26]. An association between Lphn3 loss-of-function and ADHD-like traits was discovered in Lphn3 gene knock-out animal models [27–29]. Indeed, behavioral assays detected hyperlocomotion in all animal models lacking Lphn3 gene or its orthologs, a feature reminiscent of the hyperactive trait observed for ADHD, while molecular components of the dopamine-dependent reward pathways showed divergent but persistent differences depending on the animal model tested [27–34]. Importantly, Lphn3 gene deficient animals were responsive to psychostimulant medication used for the treatment of ADHD, thus indicating that the experimentally-created condition depending on Lphn3 expression generates important pathological aspects reminiscent of clinical manifestations of the disorder [31, 34]. While Lphn3 gene knock-out animal models retain a certain face-validity to implicate a possible role of Lphn3 loss-of-function in ADHD, they lack in construct validity since none of the known genetic modifications identified in cohorts to date represent complete gene deletion. Therefore, additional data is still lacking in order to satisfactorily establish a model that would recapitulate ADHD-related cellular defects. Nevertheless, models which would mimic the genetic makeup of ADHD are prone to better reflect the defects observed in humans. To this effect, exome sequencing identified common ADHD-related variants as single amino acids substitutions in key Lphn3 domains responsible for protein-protein interactions such as the olfactomedin and GAIN domains, but their impact on receptor function has not been investigated so far [25]. Here we investigated known non-synonymous mutations described in Lphn3 genomic sequences of ADHD-affected individuals to systematically describe cellular and molecular defects resulting from these single amino-acid substitutions. Importantly, we describe a common signaling defect unifying the etiological potential of these ADHD-related polymorphism which might single-out a pathway uniquely affected in this neurodevelopmental disorder.

## Materials and methods

### Expression constructs

#### Latrophilin missense variants

Human Lphn3 expression constructs encoding the different ADHD-related variants (Lphn3^*A247S*^, Lphn3^*R465S*^, Lphn3^*D615N*^, Lphn3^*T783M*^) and their mVenus-fused versions were generated by directed mutagenesis using pCMV-Lphn3^*HA,Flag*^ and Lphn3^*HA,Flag*^-mVenus as templates [18] respectively.

#### Teneurin and FLRT

Plasmids encoding transmembrane versions of FLRT3 and Teneurin-4 as well as soluble FLRT3^*ECD*^-Fc and Ig-Fc were previously described [12, 19]. 8xHis-tagged Teneurin-2^*ECD*^ (Ten2^*ECD*^-His) is described in Supplementary methods.

#### *BRET and FRET biosensors*

BRET-based G protein biosensors and FRET-based RhoA biosensor were described elsewhere [35, 36].

### Cell aggregation assays

Intercellular adhesion assays were performed as described previously [11]. In summary, human embryonic kidney cells (HEK293) were transfected with indicated expression vectors and detached after 48 h using 1 mM ethylene glycol-bis (β-aminoethyl ether)-N,N,N′,N′-tetra acetic acid (EGTA; Sigma Aldrich) in Phosphate buffer saline (PBS; Corning). Cell populations were mixed accordingly and incubated under gentle agitation at room temperature in Dulbecco’s modified Eagle medium (DMEM; Corning) containing 10% fetal bovine serum (Biowest, France) and (in mM): 50 Hepes-NaOH, pH 7.4, 10 CaCl_2_, 10 MgCl_2_. Cell aliquots were monitored at indicated time intervals by spotting samples onto culture slides and imaging by epifluorescence microscopy using an upright Nikon Eclipse 80i microscope equipped with a 10× objective coupled to a Nikon sight-DG-Ri1 acquisition camera and Axion software (1024 × 1024 pixels resolution). The resulting images were then analyzed by counting the number and area of individual particles in the fields of view using NIS-Elements AR version 3.1 and ImageJ software version 1.5. An experimentally determined value for particle area from non-aggregated samples (negative controls) was set as the lower threshold. The aggregation index was obtained by calculating the sum area occupied by particles surpassing the determined threshold expressed as a fraction of total particle area in a given field of view.

### Saturation ligand binding assays

This assay was essentially performed as described elsewhere [11]. 24 h post-transfection, HEK293 cells expressing the respective receptors were transferred into 96-well plates (50,000 cells/well) and cultured for an additional 24 h. Cells were incubated overnight at 4 °C with increasing concentrations of the corresponding ligands (Fc- or His-tagged proteins) in DMEM containing 0.01% BSA and (in mM): 50 Hepes (pH 7.4), 2 CaCl_2_, 2 MgCl_2_. Cells were washed once to remove unbound ligands and fixed with 4% paraformaldehyde in PBS for 10 min on ice. Fixed cells were then incubated in blocking solution (3% BSA in PBS) before adding horseradish peroxidase (HRP)-coupled antibodies such as: (a) anti-human IgG-Fc antibody (1: 32,000; ThermoFisher Scientific, A18817) for FLRT3^*ECD*^-Fc assays or (b) anti-His tag antibody (1: 2000; Cell Signaling Technology, 12688) for Ten2^*ECD*^-His assays. Surface-bound ligand was detected by a colorimetric assay consisting of the conversion of peroxidase substrate 3,3′,5,5′-Tetramethylbenzidine (TMB; Sigma Aldrich) from a blueish solution to a yellowish solution after addition of HCl 2 N. Absorbance readings were obtained with the Nanoquant Infinite M200 (Tecan) microplate reader using the Tecan i-control v1.9 software. Specific binding was obtained by subtracting the absorbance values at 450 nm obtained in mock-transfected cells samples.

### Detection of cell surface receptor expression (DECS assays)

24 h post-transfection, HEK293 cells expressing the respective receptors were transferred to 24-well plates (2.5 × 10^5^ cells/well) and cultured for an additional 24 h. Cells were washed with cold PBS and fixed with 3.7% formaldehyde for 10 min. Fixed cells were then incubated in blocking solution (5% milk in TBS) for 30 min before adding anti-Flag antibody (1: 3000; Sigma Aldrich, F7425) for 1 h at room temperature. Cells were washed twice with TBS, then an anti-rabbit IgG HRP-linked antibody was added (1: 3000; Cell Signaling, 7074 S) and incubated for 30 min at room temperature. Addition of TMB substrate to each well allowed for absorbance readings at 450 nm, using the Nanoquant Infinite M200 microplate reader, after stopping the enzymatic reaction with HCl 2 N. Values obtained for mock-transfected cells were subtracted from absorbance measurements obtained from wells containing receptor-expressing cells to determine the resulting specific signal.

### Immunofluorescence and staining procedures

#### Cell surface labelling assays

This assay was essentially performed as described elsewhere [11]. HEK293T cells transfected with the indicated expression vectors were incubated in DMEM containing 0.1% BSA and 20 mM Hepes, pH 7.4 and 0.15 μM Ig fusion protein for a period of 16 h at 4 °C with gentle agitation. Excess Fc-proteins was removed, and cells were fixed with 4% paraformaldehyde for 10 min on ice. A 15 min blocking step was followed by incubation with rabbit anti-Flag antibody (1: 200; Sigma Aldrich, F7425) and Alexa Fluor-coupled secondary antibodies (anti-human IgG Alexa Fluor 488 and anti-rabbit IgG Alexa Fluor 633; ThermoFisher Scientific, A11013 and A21070) for 1 h each at room temperature. Finally, nuclear staining was conducted by incubating the cells in a 300 nM solution of 4′,6-diamidino-2-phenylindole (DAPI; ThermoFisher Scientific) for 5 min at room temperature before specimen mounting on microscope slides.

#### Filamentous actin staining assays

Transfected HEK293T cells were washed once with PBS and fixed with 4% paraformaldehyde for 15 min on ice. Fixed cells were permeabilized with 0.1% Triton X-100 at room temperature for 7 min, washed with cold PBS and incubated with a phalloidin-rhodamine:PBS solution (1: 150; ThermoFisher Scientific) for 1 h at room temperature in the dark followed by DAPI staining.

### BRET-based G protein biosensor activity assays

Receptor-mediated constitutive activity assays were conducted by co-transfecting HEK293 cells (3.5 × 10^5^ cells/transfection samples) with increasing amounts of receptor plasmid DNA (as indicated in figures) along with G protein biosensors expression vectors as per the following scheme: 40 ng of RlucII-Gα (Gα12, 13, q, i1, i2, i3 and s correspondingly), 250 ng of Gβ1 and 250 ng of GFP10-Gγ1, a total of 1000 ng was completed in each case using empty pCMV vector DNA. 48 h post-transfection, cells were incubated in BRET buffer (in mM: 10 Hepes, 1 CaCl_2_, 0.5 MgCl_2_, 4.2 KCl, 146 NaCl, 5.5 glucose, pH 7.4) and luciferase activity was induced by the addition of 5 μM coelenterazine-400A (GoldBio). For co-culture assays, 6 × 10^4^ Lphn3 read-out cells expressing biosensors (as described above) were mixed with 4 × 10^4^ HEK293 cells transfected with FLRT3 or Teneurin-4 or pCMV constructs (inducer cells) at indicated pre-reading times and allowed to adhere to the bottom of 96-wells white plates before being incubated in BRET buffer in the presence of 5 μM coelenterazine-400A. For all BRET^2^ measurements, endpoint readings were initiated after 5 min equilibrium using multimode readers TriStar^2^ (Berthold) or Cytation 5 (Biotek). BRET^2^ ratios were determined by calculating the ratio between acceptor GFP10 fluorescence emission (515/40 nm) and donor RucII luminescence emission (410/80 nm). Biosensor validation assays involved ligand-dependent GPCR activation with the following compounds: Angiotensin II (Phoenix Pharmaceuticals), nociceptin (Enzo Life Sciences), isoproterenol (Millipore Sigma) and U-46619 (Tocris/Bio-Techne). For practical purposes, an inverse BRET (iBRET) index was liberally implemented as follows in order to couple increasing index values to increasing activation profiles of the biosensors: BRET_(0)_/BRET_(x)_, where variables represent the BRET ratio signal at 0 and at x ng of receptor plasmid DNA respectively, denoting activation when values show significance above 1.

### FRET-based RhoA biosensor activity assays

HEK293 cells (3.5 × 10^5^) were transfected with 10 ng of RhoA biosensor expression plasmid and 750 ng of corresponding variant receptor-encoding plasmid DNA or empty vector pCMV and equally distributed into ten wells of 96-wells black plates. 48 h post-transfection, cells were incubated in BRET buffer and analyzed by fluorescence emission spectrum scanning on Cytation 5 microplate reader (BioTek), using an excitation wavelength of 420/30 nm and capturing the resulting emission spectra from 460/10 to 600 nm with 3 nm reading intervals. Background emission spectra obtained from empty vector-transfected cells were subtracted and the FRET ratio was determined by calculating the normalized YFP/CFP emission ratio.

### Statistical analysis

No pre-specified effect size was established as samples were randomly collected until reaching statistical power. Data are expressed as means ± standard error of means (SEM) of at least three independent experiments. Statistical analysis was performed with GraphPad Prism version 6.0 using one-way ANOVA or two-way ANOVA followed by a Dunnett’s or Sidak test. *P* values are indicated in figure legends.

## Results

### Receptor expression levels and high-affinity heterophilic interactions are maintained in Lphn3 variants harboring extracellular ADHD-related missense mutations

**Fig. 1:**
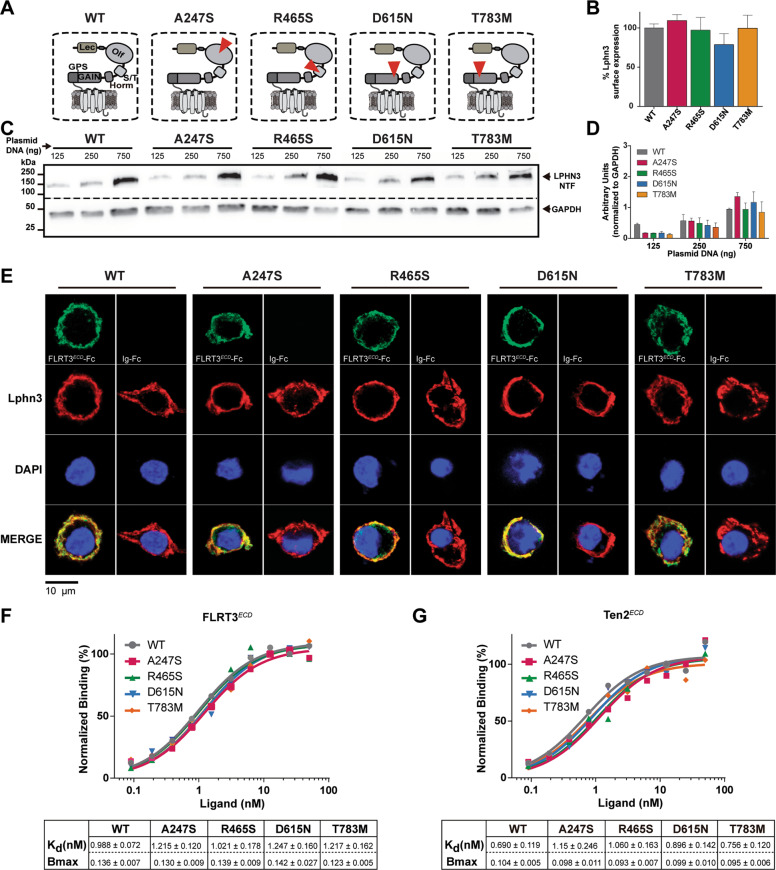
ADHD-related missense variants retain Lphn3 expression pattern and high-affinity interaction parameters for FLRT3 and Teneurin-2 ligands. **A** Schematic representation of Lphn3 domain structure and organization with corresponding ADHD-associated missense mutations identified in each dashed box (red triangle). Domain legends: Lec, Lectin; Olf, Olfactomedin; S/T, serine - threonine; Horm, hormone binding domain; GAIN, GPCR autoproteolysis-inducing domain; GPS, GPCR proteolysis site. **B** Detection of cell surface exposed N-terminal Flag epitope from ADHD-related Lphn3 variants expressed in percentage of Lphn3 WT signals. Data are represented as the mean values obtained from at least three independent experiments (*n* = 3); see *Methods* section for error bars description. **C** Immunoblotting of total cell lysates from cells transfected with increasing amounts of plasmid DNA encoding Lphn3 and ADHD-related Lphn3 variants, detecting receptor-fused N-terminal Flag epitope and endogenous GAPDH as loading control. **D** Quantification of the normalized data obtained from (**C**), data are represented as the mean values obtained from at least three independent experiments (*n* = 3); see *Methods* section for error bars description. **E** Cell surface labelling assay displaying indicated receptor-expressing cells visualized using anti-Flag antibody (red fluorescent signal from secondary antibody coupled to Alexa 633) and cell surface-bound FLRT3^*ECD*^-Fc ligand detected with an anti-human IgG antibody (green fluorescent signal from secondary antibody coupled to Alexa 488) in comparison to negative control conditions depicting the absence of signal when cells where incubated with adjunct Ig-Fc protein alone. **F**, **G** Normalized saturation binding assays conducted with ligands FLRT3^*ECD*^-Fc (**F**) or Ten2^*ECD*^-His (**G**) with cells expressing indicated receptor variants displaying respective affinity parameters (Kd) and data for maximum expression levels expressed as Abs_450_ values (Bmax). Data are represented as the mean values obtained from at least three independent experiments (*n* = 3).

Five coding polymorphisms of the Lphn3 gene were described in samples from ADHD patient groups with a high prevalence of the disorder [25]. Among them, four missense mutations A247S, R465S, D615N, and T783M modify the extracellular region of the receptor known to bear protein-protein interaction domains mediating intercellular contacts (Fig. 1A). Given that mutations can lead to propagating conformational anomalies which often spread to multiple domains of a given receptor and can result in a cascade of collateral defects, we analyzed net protein expression which effectively represents the balance between degradation and synthesis, thus can be used as a criterion to monitor protein structural stability. Therefore, we sought to verify the expression of Lphn3 receptor variants by immunodetection of the N-terminal Flag epitope in whole-cell lysates. We found no significant differences in expression levels between Lphn3-WT and receptor variants (Fig. 1C, D). As membrane compartmentalization is an important feature of GPCRs and is dependent on proper receptor folding, we quantified the detection of cell-surface exposed Flag epitope which revealed that all receptor variants possessed similar membrane exposure as Lphn3-WT (Fig. 1B). Since the N-terminal extracellular region is directly affected by the engineered mutations and that this portion of the receptor bears the domains responsible for ligand interaction, we conducted a ligand-mediated surface labelling of these receptor variants using soluble FLRT3 extracellular domain fused to the constant fraction of human IgG (Fig. 1E and Supplementary Fig. 1A, B). An immunofluorescent signal for FLRT3^*ECD*^-Fc was detected at the surface of cells individually expressing each receptor variant and not on naive cells (Fig. 1E and Supplementary Fig. 2), suggesting that ligand-receptor interactions were not disrupted by the introduction of the different mutations. This led us to determine the receptors’ affinity constant toward their ligands, a crucial parameter of ligand-receptor interaction which would allow us to detect possible changes in conformational states of the binding pocket as a result of introduced mutations. Saturation binding assays conducted using soluble FLRT3 or Teneurin-2 as ligands revealed that all receptor variants maintained a high-affinity profile comparable to their wildtype counterpart as denoted by Kd values and confirmed similar cell-surface expression levels according to Bmax values (Fig. 1F, G and Supplementary Fig. 3A–J). Together, these data suggest that receptor availability and intermolecular factors governing Lphn3’s propensity to produce ligand-mediated intercellular adhesion are both maintained in the presence of ADHD-related mutations.

### Lphn3 ADHD-related missense mutations impede receptor-mediated intercellular adhesion

**Fig. 2:**
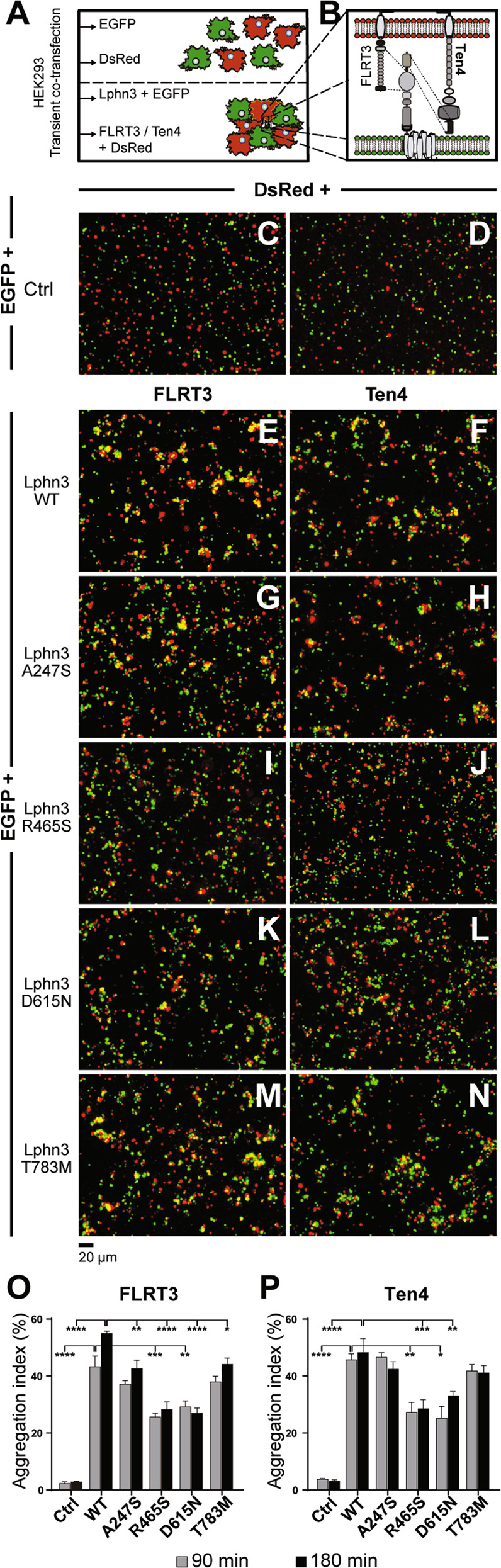
Heterotypic intercellular cell adhesion mediated by Lphn3 is hindered by the presence of NTF missense mutations associated with ADHD. **A** Illustration of the experimental setup used for cell aggregation assays. **B** Schematic representation of the molecular configuration taking place in aggregation assays between Lphn3 and its transmembrane ligands FLRT3 and Teneurin-4, depicting the respective non-overlapping interaction sites with dash lines connecting the adhesion domains involved. **C**–**N** Representative epi-fluorescent microscopy images with full field of view displaying cell aggregates at 180 min between indicated receptor-expressing cells co-transfected with EGFP (green puncta) and FLRT3- or Ten4-expressing cells co-transfected with DsRed (red puncta) or negative control conditions containing cells expressing only DsRed mixed with EGFP-expressing cells. **O**, **P** Aggregation index of data obtained in (**C**–**N**) for FLRT3 and Ten4 respectively. *P* values describing significance between receptor variants and Lphn3 positive control: *P < 0.05, **P < 0.01, ***P < 0.001, ****P < 0.0001. Data are represented as the mean values obtained from at least three independent experiments (*n* = 3); see *Methods* section for error bars description.

Lphn3-mediated intercellular adhesion confers synapse specificity through heterophilic interactions with its transmembrane ligands FLRT and Teneurin [16]. In order to determine the impact of these mutations on Lphn3 adhesion function, we conducted cell aggregation assays which consisted in co-culturing two cell populations independently expressing Lphn3 receptor variants or their adhesion-competent ligands (Fig. 2A). Upon contact between both cell populations, heterophilic interactions between Lphn3 receptors and their ligands will occur and stabilize cell-cell contacts, thus forming aggregates (Fig. 2A, B) [11, 12]. FLRT3 and Teneurin-4 were assayed independently and selected on the basis of their non-overlapping binding interfaces with Lphn3; FLRT3 for its essential interaction with Lphn3 olfactomedin domain and Teneurin-4, known to mainly establish molecular contacts with the receptor’s lectin domain (Fig. 2B). Control conditions assaying cells intracellularly expressing green and red fluorescent proteins displayed a pattern of random cell distribution characteristic of non-interacting cell populations (Fig. 2C, D). In contrast, positive control conditions consisting of Lphn3-expressing cells in contact with FLRT3- and Ten4-expressing cells revealed the formation of large aggregates (Fig. 2E, F). Heterotypic intercellular adhesion was observed for all receptor-containing samples in presence of both ligands reaching a plateau at 90 min (Fig. 2E–P), although the aggregation index was significantly lower for all variants in contact with cells expressing FLRT3 in comparison to Lphn3-WT (Fig. 2O), whereas a similar decrease was observed for R465S and D615N variants in contact with cells expressing Teneurin-4 (Fig. 2J, L, P). Although indicating that the integrity of the NTF is required for stabilizing cell-cell adhesion through heterophilic interactions, these results were surprising given that all missense mutations tested, except for A247S, modify receptor domains that have not been identified as being part of Lphn-ligand interaction interfaces. Thus, the data indicate that Lphn3-mediated intercellular adhesion events are not strictly dependent on extracellular Lphn-ligand interactions but rather involve ligand-independent intrinsic receptor properties.

### G protein signal profiling of ADHD-related Lphn3 receptor variants unveils a selective coupling deficiency toward Gα13

**Fig. 3:**
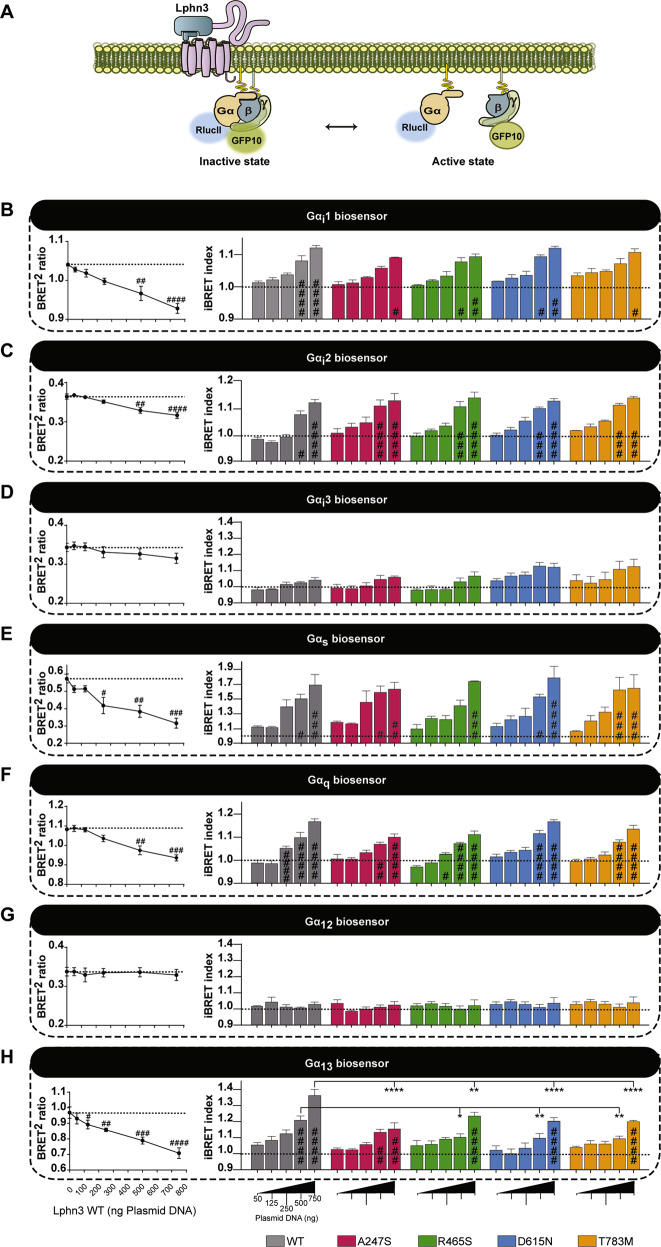
G13 signaling pathway is selectively impaired by intrinsic activation properties of ADHD-related Lphn3 variants. **A** Schematic representation of intermolecular G protein inversion-BRET biosensors depicting inactive conformations characterized by the presence of high BRET^2^ signal due to spatial proximity between RlucII and GFP10, and active conformations leading to low BRET^2^ signal following structural rearrangements resulting in the distancing of RlucII from GFP10. **B**–**H** Representative Lphn3-WT BRET^2^ ratio curves (*left panels*) followed by inversion-BRET biosensors activation profile represented by the iBRET index (*right panels*) for all variants, in relation to increasing receptor expression levels as a result of increasing DNA concentrations (0–750 ng) and assessing their intrinsic ability to induce functional coupling with: Gi1 (**B**), Gi2 (**C**), Gi3 (**D**), Gs (**E**), Gq (**F**), G12 (**G**) and G13 (**H**). Data are represented as the mean values of at least three independent experiments of four replicates each (*n* = 3); see *Methods* section for error bars description. *P* values describing significance between receptor variants and Lphn3 control: *P < 0.05, **P < 0.01, ***P < 0.001, ****P < 0.0001; *P* values describing significance between 0 ng DNA (dotted line) and a given DNA concentration within the indicated receptor variant group: ^#^*P* < 0.05, ^##^*P* < 0.01, ^###^*P* < 0.001,^####^*P* < 0.0001.

Intrinsic properties describing the function of Lphn3 include its ability to constitutively couple to various subfamilies of G proteins [22]. Therefore, we adopted a strategy enabling us to systematically dissect Lphn3’s G protein coupling efficiency with select G protein families by the use of molecular tools relying on bioluminescence resonance energy transfer (BRET). G protein BRET-based biosensors were selected and assayed with various well-characterized GPCRs in order to validate their dynamic range to produce a BRET signal (Supplementary Fig. 4A–G). This signal ratio was liberally converted into an iBRET index to describe activation profiles of biosensors proportionally with increasing values. Our approach consisted in co-expressing receptor variants in HEK293 cells along with BRET biosensors capable of detecting conformational changes following activation unveiled by the spatial distancing between RlucII-fused α and GFP10-fused γ G protein subunits (Fig. 3A). In order to set the conditions to test for constitutive activity of the receptors, different expression levels were obtained for each variant by increasing receptor-encoding plasmid DNA concentrations during transfection assays while keeping biosensors constant throughout. While assaying members of the Gi family biosensors, we found that Lphn3-WT and its variants displayed similar efficiency in coupling to all Gi biosensors and that they demonstrated a higher activation kinetic when coupled to Gi1 and Gi2 with an almost undetectable Gi3 iBRET signal (Fig. 3B–D and Supplementary Fig. 5A–C). Subsequently, we probed the activation of a Gs biosensor which revealed that Lphn3 constitutively induced a robust increase in iBRET signal and that this activity was unaffected by the presence of ADHD-related mutations (Fig. 3E and Supplementary Fig. 5D). We then focused on assaying G protein biosensors which relay the activity of G proteins known for mediating GPCR signaling toward actin cytoskeleton components such as Gq and G12/13 (Fig. 3F–H and Supplementary Fig. 5E–G). An appreciable iBRET signal was detected with the Gq biosensor when assaying the constitutive activity of Lphn3 and all receptor variants, denoting indistinguishable activation levels among all variants and Lphn3-WT receptor (Fig. 3F and Supplementary Fig. 5E). Biosensors of the G12/13 families were tested separately in order to further dissect the distinct coupling of this functionally related pair of G proteins. Surprisingly, while Lphn3-WT did not display a detectable constitutive activation of the G12 biosensor, it did produce a robust functional coupling to G13, thus unveiling the receptor´s selective coupling profile to this G protein subfamily (Fig. 3G, H and Supplementary Fig. 5F,G). ADHD-related receptor variants were similarly unable to generate an intrinsic activation of the G12 biosensor but did induce a modest activation of the G13 biosensor, suggesting that preferential coupling selection was unchanged amid the presence of missense mutations (Fig. 3G, H). However, the activation magnitude reached by each receptor variant was significantly reduced (~50%) when compared to control Lphn3-WT conditions. Taken together, these data suggest that the presence of missense mutations associated with ADHD gives rise to a selective impairment in the intrinsic G protein coupling properties of the Lphn3 receptor uniquely targeting the actin cytoskeleton-associated G13 protein signaling.

### Lphn3-mediated actin remodeling is altered by ADHD-related polymorphism A247S and R465S

**Fig. 4:**
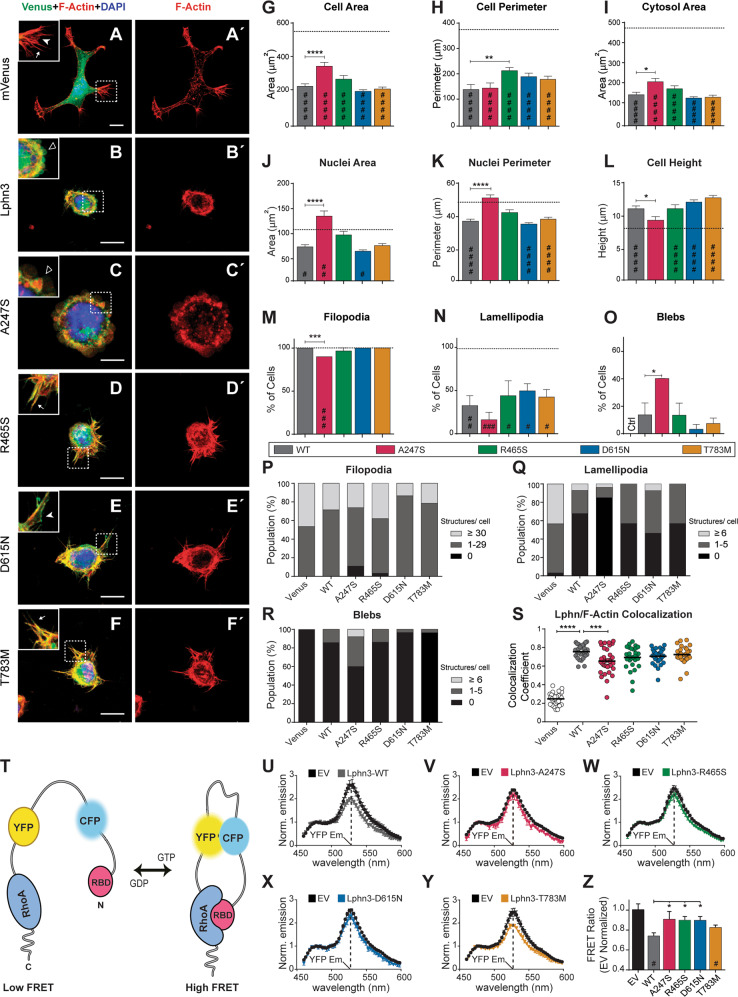
Lphn3-dependent actin cytoskeleton remodeling is altered in the presence of ADHD-associated A247S and R465S mutations while Lphn3-mediated RhoA activity regulation is additionally affected by D615N mutation. **A**–**F** Representative confocal microscopy images depicting the merged signal for indicated mVenus-fused receptor variants (in green), F-actin (in red) and nuclei staining (in blue). Coincident green and red signals are visualized as yellow pixels. Insets indicate the following actin-dependent structures: filopodia (arrows), lamellipodia (white arrowheads) and blebs (empty arrowheads). Scale bar: 10 μm. **A**’–**F**’ Images corresponding to (**A**–**F**) depicting F-actin staining only. **G**–**L** Quantification of data related to individual cell area, cell perimeter, cytosol area, nuclei area, nuclei perimeter and cell height, respectively, for cells expressing the indicated receptor variant. **M**–**O** Quantification of cell population harboring the indicated actin-dependent extensions for cells expressing the corresponding receptor variants. Note that blebs are absent from control cell conditions (Ctrl) shown in (**O**). **P**–**R** Percentage distribution of cell populations harboring the indicated number interval of actin-dependent structures for indicated receptor-expressing cell samples. **s** Pearson’s coefficient quantification describing colocalization between F-actin fluorescent signal and fluorescence emitted by indicated mVenus-fused receptor variants or mVenus-only negative control. Data in (**G**–**S**) are represented as the mean values of at least three independent experiments (Ctrl *n* = 28, WT *n* = 28, A247S *n* = 27, R465S *n* = 29, D615N *n* = 30, T783M *n* = 28). **T** Schematic representation of the ratiometric FRET-based RhoA biosensor comprising full length RhoA N-terminally fused in tandem to yellow-fluorescent protein (YFP) and cyan-fluorescent protein (CFP) followed by the Rho-Binding Domain of the effector rhotekin (RBD). Active GTP-bound RhoA will bind to its effector domain RBD, thus leading to the biosensor undergoing a conformational change and positioning YFP and CFP in close proximity. Conversely, RhoA GDP-bound form is prevented from binding to RBD and therefore increases the distance between YFP and CFP. High FRET ratio measurements, represented by high emission intensity at YFP wavelength through initial CFP excitation protocol, will denote RhoA activation while low FRET ratios denote RhoA inactivation. Note that the freely accessible C-terminal of RhoA in this biosensor preserves its potential for inactivation by endogenous Rho-GDP Dissociation Inhibitor proteins. **U**–**Y** Representative FRET-induced emission spectra of the RhoA biosensor to monitor RhoA activity through YFP/CFP ratiometric emission intensity in the presence of Lphn3-WT and ADHD-associated variants. **Z** Normalized ratiometric YFP emission intensity of the FRET-based RhoA biosensor in the presence of Lphn3-WT and ADHD-associated variants. Data in (**Z**) are represented as mean values of at least three independent experiments; see *Methods* section for error bars description. Dotted black lines shown in (**G**–**N**) represent mean values observed for negative control mVenus-expressing cells. *P* values describing significance between receptor variants and Lphn3 control: *P < 0.05, **P < 0.01, ***P < 0.001, ****P < 0.0001; *P* values describing significance between negative control mVenus or empty vector (EV)-expressing cells and a given receptor variant: ^#^*P* < 0.05, ^##^*P* < 0.01,^###^*P* < 0.001, ^####^*P* < 0.0001.

The actin cytoskeleton acts in conjunction with adhesion molecules to stabilize cell-cell or cell-matrix contact junctions through the formation of morphologically defined structures decorating the cell periphery. Given the selective alteration of actin cytoskeleton-linked G13 signaling observed for Lphn3 variants amidst a robust activation of Gq and Gi proteins, we investigated the intrinsic ability of the receptor variants to influence the previously described Lphn3-mediated actin cytoskeleton remodeling in mammalian cells [18]. Thus, Lphn3 receptor and missense variants fused C-terminally to mVenus were overexpressed in HEK293T cells which were later submitted to fluorescent staining of filamentous actin (F-actin) and nuclei in order to analyze morphological parameters and the formation of actin-dependent cellular structures (Fig. 4A–F, A’–F’). Consistent with a role in actin cytoskeleton signaling, images captured using confocal microscopy revealed a decrease in the area and perimeter of Lphn3-expressing cells when compared to control cells expressing mVenus alone (Fig. 4G, H). This reduction pattern was preserved for cells expressing each receptor variant except for A247S-expressing cells which displayed a less severe attenuation of these parameters resulting in cells harboring larger areas (Fig. 4G). Another exception was the R465S-expressing cells which harbored a larger perimeter without increasing their area, suggesting the presence of membrane structures with low surface area. The effect of Lphn3 expression on cell nuclei parameters was reversed in cells expressing A247S, which was not the case for cells expressing the remaining receptor variants (Fig. 4J, K). Consequently, the cytosol area observed for A247S-expressing cells was significantly larger than for other Lphn3-expressing cells (Fig. 4I). A concomitant decrease in cell height was measured for A247S-expressing cells when compared to the remaining groups of Lphn3-expressing cells which displayed the vertical amplification that is characteristic of Lphn expression in HEK293T cells [18] (Fig. 4L). Subsequently, we focused on monitoring the formation of actin-dependent cell extensions to evaluate the effect of receptor variant expression on the stabilization of such structures. For this, we characterized morphologically identifiable F-actin structures (filopodia, lamellipodia or blebs) from transfected HEK293T cells taken in isolation to allow for undisturbed whole-cell analysis in conditions which maximized cell-matrix contacts (Fig. 4A’–F’). Once analyzed in comparison to Lphn3-expressing cells, other features specific to A247S and R465S were detected. We observed a decrease in the number of filopodia and lamellipodia extending from A247S-expressing cells as evidenced by cells having the lowest filopodia and lamellipodia content in terms of proportion of cells harboring these structures (Fig. 4M, N) and their number per cell (Fig. 4P, Q). In contrast, cells expressing the A247S variant presented more blebbing events as observed by an increase in cell population harboring blebs (Fig. 4O) as well as the increased number of blebs per receptor-expressing cells (Fig. 4R). As reported previously, control HEK293T cells did not present blebbing in normal growth conditions but did so only when expressing Lphn (Fig. 4B, C, O, R) [18]. Expression of the R465S variant potentiated filopodia formation with a significant increase in the cell population harboring the highest number of these actin-dependent structures (Fig. 4P). Finally, we sought to evaluate the extent of receptor/F-actin colocalization using Pearson coefficient values as an indicator of the degree of complex stabilization with the actin cytoskeleton as previously reported [18]. While a high F-actin colocalization index was detected for all receptor variants, the A247S variant significantly decreased receptor coincident labeling with this cytoskeletal protein as compared to Lphn3 WT receptor (Fig. 4S). On the other hand, F-actin content and density was significantly increased in R465S-expressing cells (Supplementary Fig. 6). These data reveal that the actin cytoskeleton signaling events elicited by Lphn3 are impeded by the expression of A247S and R465S receptor variants in a non-overlapping manner, supporting an altered functional coupling to intracellular effectors linked with actin cytoskeleton signaling.

### The activity of actin cytoskeleton modulator RhoA is inefficiently regulated by Lphn3 bearing ADHD-related polymorphisms A247S, R465S and D615N

The small GTPase RhoA is intimately linked to the modulation of actin cytoskeleton signaling and is differentially regulated by distinct G protein families promoting either an activation through Gi/Gq/G13 or an inhibition through the Gs pathway. Since Lphn3 signaling revealed a broad G protein coupling profile linked to both RhoA-activating and RhoA-inhibiting functions, we investigated the activity of RhoA as an indicator of the net integrated signals incoming from endogenous G protein signaling. To achieve this, we implemented the use of a well-characterized ratiometric FRET-based RhoA biosensor which is responsive to both inhibitory or activating signals (Fig. 4T) [36]. As observed in initial reports, this biosensor displayed a certain level of basal activity when expressed alone in HEK293 cells (Fig. 4U–Y; Empty vector condition). Through the determination of the RhoA activation profile, we detected that Lphn3-WT constitutive signaling led to a significant inhibition of the biosensor’s activity and to a lesser extent so did the T783M receptor variant (Fig. 4U, Y, Z). However, receptor variants A247S, R465S and D615N displayed a lack of RhoA inhibition (Fig. 4V–X, Z). These data unveil the inability of Lphn3 ADHD-related variants to properly modulate RhoA activity, a key determinant of actin cytoskeleton signaling.

### A synergistic transcellular FLRT3/G13 signaling deficiency undermines the function of Lphn3 bearing ADHD-related mutations

**Fig. 5:**
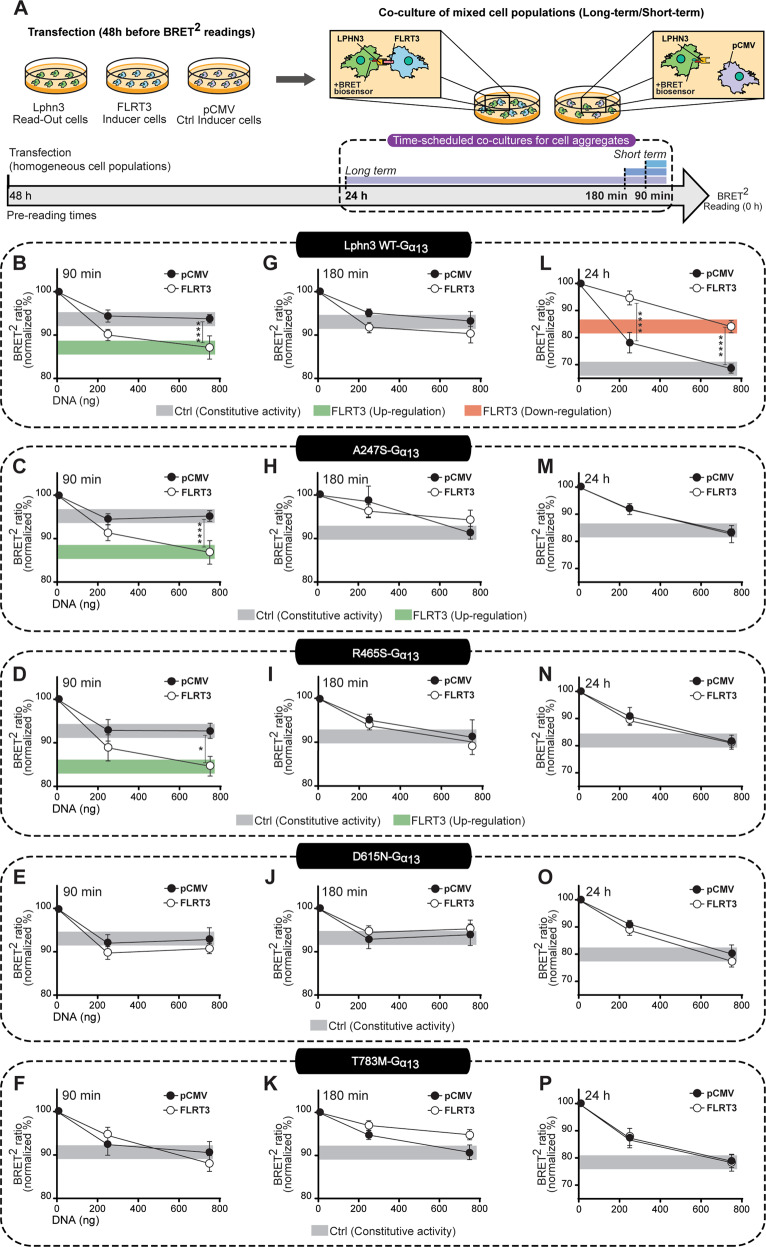
Aberrant transcellular signaling elicited by ADHD-associated Lphn3 variants through FLRT3-mediated modulation of G13 activity in intercellular adhesion events. **A** Workflow diagram for scheduled co-culture experiments culminating in BRET measurements. Read-out cells expressing G13 BRET-based biosensor along with Lphn3-WT or its variants were mixed with inducer cells transfected with FLRT3 or empty vector pCMV and co-cultured according to a long-term scheme (24 h) or short term schemes (90 and 180 min) before being monitored for the presence of BRET signal 48 h post-transfection. **B**–**P** BRET^2^ ratio detected in read-out cells represented as the percentage of maximum BRET^2^ ratio values obtained in the absence of receptor expression (100%) when cells were co-cultured with pCMV-transfected cells or FLRT3-expressing inducer cells during a short-term period of 90 min (**B**–**F**) and 180 min (**G**–**K**) or a long-term period of 24 h (**L**–**P**). Receptor-mediated constitutive activity of the G13 BRET-based biosensor at 750 ng of respective receptor plasmid DNA is indicated by *gray boxes* while up-regulation or downregulation of biosensor activity in relation to constitutive activity is indicated by *green boxes* or *red boxes* respectively. Data are represented as mean values of at least three independent experiments; see *Methods* section for error bars description. *P* values describing significance between the conditions corresponding to co-cultures with FLRT3-expressing inducer cells and pCMV-transfected cells: *P < 0.05, **P < 0.01, ***P < 0.001, ****P < 0.0001.

Apart from exerting ultrastructural functions directed at physically adjoining cell membranes, transmembrane Lphn ligands are also thought to exert modulatory effects on the receptors’ signaling cascades [19, 24]. FLRT3 modulation of Lphn signaling is of particular interest given that it instructs important neuronal migratory guidance cues during cerebral cortex development [37, 38]. Because intercellular adhesion events mediated by FLRT3 expression exhibited deficits across all ADHD-related Lphn3 receptor variants and considering the intrinsic G13 coupling deficiency expressed by these variants, we sought to elucidate the modulation of G13 protein signaling pattern emanating from such ligand-dependent adhesive junctions by implementing a hybrid assay involving matrix-plated cell aggregation samples (Fig. 5). Cells expressing individual Lphn3 receptor variants along with the G13 protein BRET-based biosensor (read-out cells) were co-cultured with FLRT3-expressing cells (inducer cells) and the BRET^2^ ratio was quantified at various time points so as to monitor short-term as well as long-term cell-cell contacts reminiscent of transcellular interactions suspected to be involved in early migratory events which will later be converted into more permanent junctions. Control conditions involved Lphn3-expressing read-out cells cultured alongside empty vector-transfected cells (Fig. 5A). Maximal normalized BRET^2^ ratio values (set at 100%; Fig. 5) were obtained from samples in which read-out cells lacking Lphn3 expression were mixed with empty vector or FLRT3 inducer cells. It is noteworthy that this experimental paradigm resulted in low but significant constitutive activity for short co-culture time points while displaying high constitutive activity for longer co-culture times. Nonetheless, early contact monitoring detected a FLRT3-dependent activation of the G13 biosensor which amounted to an up-regulation of the BRET ratio from the constitutive signal present for Lphn3-WT receptor, a feature that was conserved for A247S and R465S variants (Fig. 5B–D). However, FLRT3 intercellular contacts did not induce an up-regulation of the G13 biosensor activity elicited by D615N and T783M variants (Fig. 5E, F). Sustained contacts displayed an intermediary phase at 180 min during which FLRT3-induced up-regulation of G13 constitutive activity subsided for A247S and R465S receptor variants similarly to Lphn3-WT (Fig. 5G–K). This was followed by a FLRT3-induced long-term downregulation of G13 biosensor activity as compared to constitutive activity into Lphn3-WT expressing cells (Fig. 5L). Strikingly, this downregulation was not observed for any of the ADHD-related Lphn3 receptor variants, as levels of G13 biosensor activity were indistinguishable between FLRT3 and control conditions, thus accounting for the most drastic ligand-mediated signaling deficiency (Fig. 5M–P). In contrast, biosensors from the Gi, Gs or Gq families did not exhibit downregulation in response to FLRT3, indicating that FLRT3 modulation of Lphn3 signaling was predominantly targeting G13 pathway (Supplementary Fig. 7). In addition, the same assay conducted in the presence of Teneurin4-expressing cells did not detect any long-term changes in read-out cells for G13 biosensor constitutive activity nor other G protein biosensor tested (Supplementary Fig. 8). Together, these data reveal that Lphn3 ADHD-related receptor variants bear a long-term ligand-dependent modulatory deficiency centered around a FLRT3/G13 synergistic relationship.

## Discussion

A mechanistic approach in describing ADHD-susceptibility signaling pathways proved challenging due to the polygenic nature of the disorder. Our study provides evidence of a concordant dysfunction shared among distinct ADHD-related missense variants of ADGRL3/Lphn3, supporting a disease-relevant susceptibility molecular axis. Our systematic characterization of four inheritable genetic polymorphisms identified in a well-established ADHD cohort [25] highlights the difficulty in conducting functional validation studies of pathogenesis risk genes without resorting to drastic loss-of-function approaches described in gene deletion studies. Dissecting both the signaling and adhesion properties of Lphn3 ADHD risk variants we found a common but highly selective signaling impairment resulting in divergent cellular defects accompanied by partially overlapping ligand-dependent alterations of intercellular adhesion profiles. These observations unveil the subtle yet specific nature of phenotypes caused by coding polymorphisms affecting ADHD-related genes. Previously, approaches relying on the identification of defects affecting canonical protein functions yielded inconclusive results as illustrated by the study of ADHD-linked gene encoding cadherin-13, while decades of conflicting reports on the pathophysiological role of D4 dopamine receptor copy-number variants were resolved by looking at non-canonical functions [39–41]. Similarly, testing of the Lphn3-A247S mutation located at the FLRT ligand binding interface failed to detect binding defects, a result that was also corroborated by this study [42, 43]. Added to the fact that the R465S variant was identified both in control and ADHD patients, our findings are in line with the current knowledge that neuropsychiatric disorders are etiologically defined as possessing a polygenic nature with rare genetic lesions predicted to contribute small effect sizes while collectively amounting to severe symptoms affecting cognitive abilities, mood or behavior [25, 44–46].

The dysfunctional G13 coupling property described in this study as a coherent characteristic for distinct ADHD-related Lphn3 receptor variants strongly supports their pathophysiological role in constituting an important disease-relevant susceptibility axis that heavily involves an underlining deficiency in regulatory mechanisms toward actin cytoskeleton remodeling. Indeed, data-mining pathway analysis related to ADHD pointed to the disruption of many cytoskeletal elements which could be setting the tone for an interruption of age-appropriate neurodevelopmental features [47–49]. Among those affected is a member of the Ras-family of small Rho GTPases, RhoA, which plays a well-characterized role in actin cytoskeleton remodeling and is essential for determining numerous neurodevelopmental steps ranging from neuronal migration to synapse maturation [50, 51]. Modulatory actions on Rho GTPases activity are exerted by guanine nucleotide exchange factors controlled by G proteins from the G12/13 family but also Gq and Gi [52–55]. Our data describing a robust Lphn3-dependent constitutive activation of G13 but also Gq and various Gi isoforms reinforces its role in actin cytoskeleton remodeling. The lack of detectable Lphn3-dependent constitutive G12 activation reported here is peculiar but corroborates a previous observation pertaining to the inability of a constitutively active mutant to increase the activity of a transcriptional reporter under G12 supplementation conditions and highlights once again a selective coupling property of these receptors toward actin-related cytoskeleton signaling [22]. Confirming this is our observations that Lphn3 can constitutively regulate cell morphology and actin-dependent structures. Of particular interest is the formation of blebs which are membrane protrusions resulting from a detachment between the cell membrane and subjacent cortical actin governed by RhoA/myosin signaling pathways coupled to an increase in intracellular Ca^2+^ concentrations and recruitment of accessory proteins [56, 57]. Interestingly, in addition to their RhoA-modulating activity and Gq-linked Ca^2+^ regulation described here and by others, latrophilins can modulate myosin recruitment to the cell membrane thus potentially destabilizing interactions with the actin cytoskeleton cortex [58]. Bleb-forming conditions can be further exacerbated by a decrease in RhoA activity or lack of RhoA activation/modulation, consistent with the observation made for cells expressing Lphn3 and T783M but also denoted by A247S receptor variant’s inaptitude to follow suit as well as D615N and R465S. Interestingly, neuronal properties emanating from RhoA modulation deficiency correlate with cognition impairments linked to the essential contribution of actin cytoskeleton assembly/disassembly dynamics in synaptic plasticity paradigms [51, 59, 60].

The case of the A247S variant is particularly puzzling since it strongly highlights the alteration of unknown factors acting concomitantly with G13 protein deficiency in affecting intercellular adhesion on the one hand and cytoskeletal rearrangements related to cell size and bleb formation on the other. The fact that these conditions are not equally met in cells expressing all ADHD-related receptor variants despite a common G13 signaling deficiency suggests that G protein-independent mechanisms may be involved, among them the ability of Lphns to directly interact with actin cytoskeleton components through intracellular motifs [18, 61, 62]. Moreover, the observation that Lphn3/FLRT3-mediated signaling through G13 during intercellular adhesion was impacted by all ADHD-related missense variants while Teneurin4-mediated cell-cell contacts were affected by a subset of these variants, suggests a differential contribution of cytoskeletal signaling/anchoring processes to ligand-specific molecular events, thus supporting the previous identification of FLRT3 as an ADHD-associated risk gene [46]. The lack of G13 signal downregulation in response to long-term FLRT3 exposure could potentially unveil a shared deficiency in desensitization mechanisms affecting ADHD-related Lphn3 variants or an inability to convert ligand-mediated signals into adequate G protein coupling, each hypothesis requiring further investigation. However, while we cannot ascertain that the loss of intercellular adhesion directly originates from a G13 signaling deficiency, the latter constitutes an important piece of information revealing probable conformational alterations of the Lphn3 variants. Nonetheless, considering that Lphn family members have been described as determinants of migration polarity in axonal growth cone structures and that their role in synapse formation is dependent on their ability to signal through G proteins, our data are consistent with a tonic and persistent detrimental effect of Lphn3 missense variants on signaling pathways instructing brain wiring [50, 63–65].

ADHD traits comorbidity profile includes other neuropsychiatric disorders, thus pointing to an overlapping molecular causation [46, 66]. Comorbid relations have also been identified between ADHD and chronic non-neurological diseases, a feature hypothesized to originate from the contribution of risk genes affecting brain functions along with peripheral tissue functions [67, 68]. It is worth noting that Lphn3 is not only expressed in brain tissues but also in select peripheral tissues [12]. Thus, in addition to playing a role as synapse organizers in neurons, Lphns have been described as important regulators of lung function and genetic risk factors for the development of asthma, a pulmonary condition which displays a significant comorbidity with ADHD [16, 65, 68, 69]. The regulation of insulin secretion from pancreatic β cells represents yet another context-specific function elicited by islet-expressed Lphns with potential repercussions on diabetes, a metabolic condition which is also comorbid with ADHD [67, 70]. Whether Lphn3 ADHD-related variants represent molecular correlates of ADHD comorbidity profile remains unclear, however their wide expression pattern may constitute additional risk factors accompanying the predicted neurological etiology.

Lphn3 tissue distribution illustrates the importance of cellular context to assess receptor function. Our study represents an attempt to circumvent strict cellular context by providing exogenous G protein expression in order to overcome disparities that could exist in regard to endogenous G protein expression between different cell types. Thus, the selective signaling impairment displayed by Lphn3 ADHD-related variants toward G13 identified in our study is unlikely to be cell-specific. As for their ability to couple to various G proteins, the same observation has been made for many GPCRs, where signaling is dependent both on spatiotemporal bioavailability and on the cell’s expression profile of signaling effectors [71, 72]. Background expression of endogenous G proteins was preserved in our study in order to maintain a tonic competitive environment which is often required to unveil the full array of GPCR functions [73, 74]. On the other hand, it is possible that the overexpression of receptors and/or biosensors in this cellular context may have led to an environment which does not reflect physiological coupling conditions, although this might be unlikely since we replicated endogenous coupling conditions for various GPCRs from different family groups using this approach. Therefore, we infer that this methodology holds promise for deciphering the signaling profile of aGPCRs and their disease-related coding variants.

Intrinsic structural flexibility constitutes a hallmark of GPCR function, and in the case of bipartite aGPCRs this is evidenced by a high degree of modulatory effects implicating a complex interplay between the extracellular adhesion motifs-bearing subunit and the transmembrane signal-transducing region [75]. The evidence that Lphn3 variants analyzed in this study display a common signaling defect despite bearing modifications affecting functionally distinct extracellular receptor domains suggests that the receptors’ extracellular regions adopt a shared conformational state with a biased impact on the seven-transmembrane signaling unit. Rearrangements of extracellular regions is known to occur intrinsically within the proteolytically-active GAIN domain of many aGPCRs including Lphns and results in the exposure of a cryptic ligand known as the tethered agonist [75]. This structural mechanism is thought to allow for inherent receptor activation without physical disassembly of NTF and CTF subunits. Consistent with a long-range disturbance mechanism, our data revealed that local ligand interactions are unaffected by extracellular domain-specific mutations. Additional studies will have to be undertaken in order to decipher the common structural rearrangement taking place in these ADHD-related missense variants in relation to G protein coupling.

Our findings provide further understanding on ADHD-related aetiopathogenesis through the identification of a FLRT3/Lphn3/G13/RhoA/actin cytoskeleton susceptibility axis. However, additional studies will be needed in order to determine if actin cytoskeleton remodeling constitutes the main pathogenicity pathway which is propagated as part of ADHD etiology and to ascertain the systemic role of Lphn3 variants in mammalian pathophysiology linked to the development of ADHD traits. Meanwhile, our study lays the ground for the future development of pharmacological approaches aimed at targeting Lphn3 receptor variants through a scheme involving compounds with biased structural recognition or biased agonism toward G13 signaling pathway as potential alternative treatment strategies for ADHD.

## Supplementary information

Supplementary Information

Supplementary Figure 1. Purification of soluble recombinant Lphn3 ligands, FLRT3 and Teneurin2.

Supplementary Figure 2. Cell surface labelling assays of HEK293T heterogeneously expressing Lphn3 receptor variants using FLRT3 extracellular domain recombinant protein.

Supplementary Figure 3. Saturation binding curves characterizing the interaction between Lphn3 ADHD-related receptor variants and recombinant ligands FLRT3 or Teneurin-2.

Supplementary Figure 4. Functional validation of BRET-based biosensors.

Supplementary Figure 5. BRET2 ratio plots for BRET-based biosensors determined in cells expressing increasing amounts of Lphn3 or its ADHD-related variants.

Supplementary Figure 6. F-actin content analysis of cells expressing Lphn3 ADHD-related variants reveals a disequilibrium in actin dynamics displayed by R465S-expressing cells.

Supplementary Figure 7. Persistent intercellular contacts between FLRT3 and Lphn3 ADHD-related variants maintain the constitutive activity elicited by the receptors on BRET-based Gα protein biosensors

Supplementary Figure 8. Persistent intercellular contacts between Teneurin-4 and Lphn3 variants do not alter the constitutive activity elicited by the receptors on BRET-based Gα protein biosensors

## Data Availability

Plasmids are made available upon request from respective labs.
