# Supplementary material for: Convergent selective signaling impairment exposes the pathogenicity of latrophilin-3 missense variants linked to inheritable ADHD susceptibility
Source: Mol Psychiatry. 2022 Apr 7;27(5):2425–38. doi: 10.1038/s41380-022-01537-3 (PMC9135631; doi:10.1038/s41380-022-01537-3)
Supplement: Supplementary file 1 — Supplementary Information [file 41380_2022_1537_MOESM1_ESM.docx]

**Supplementary information: Moreno-Salinas *et al.*** *Convergent selective signaling impairment exposes the pathogenicity of latrophilin-3 missense variants linked to inheritable ADHD susceptibility*

**Supplementary methods**

**Plasmids cloning and engineering**

***Latrophilin-3 constructs-*** Human Lphn3 expression constructs encoding the different ADHD-related variants (Lphn3*^A247S^*, Lphn3*^R465S^*, Lphn3*^D615N^*, Lphn3*^T783M^*) versions were generated by directed mutagenesis using pCMV-Lphn3*^HA, Flag^* as template ^1^. The following oligonucleotides were used: A247S (Forward: CATTGCAAACTCCAATTATC, reverse: GATAATTGGAGTTTGCAATG); R465S (Forward: GGCGCAACTCGTCTACAAG, reverse: CTTGTAGACGAGTTGCGCC); D615N (Forward: GAGGCAAGAACAGCGCCG, reverse: CGGCGCTGTTCTTGCCTC); T783M (Forward: CCTGTGATCATGGCAGCCATTA; reverse: TAATGGCTGCCATGATCACAGG). The mVenus-tagged version of these constructs was generated by replacing the EcoRI-BSU36 fragment from pCMV Lphn3*^Flag-HA-mVenus^* ^1^ with the corresponding fragment of aforementioned receptor variants plasmids. All constructs were verified by automated DNA sequencing. Although variants are named according to the original numbering reported by Domené et al ^2^ for isoform2-Swissprot Q9HAR2, revised sequence Q9HAR2-1 points to position 245 for Ala247Ser mutation (protein sequence ^241^EAIIA^245^) and to position 770 for Thr783Met mutation (protein sequence ^766^SPVIT^770^).

***Teneurin^ECD^-His construct-*** 8xHis-tagged Teneurin-2*^ECD^* (Ten2*^ECD^*-His) was generated by PCR amplification of the sequence corresponding to Teneurin-2 extracellular region (aa Ala^392^-Gly^2772^; Accession number: XP_016865149.1) from pcDNA3-LassoB*^Flag-^*^Myc^ ^3^ using an antisense oligonucleotide inserting a C-terminal octa-histidine tag as follows: forward: TATAGCTAGCGATTACAAGGATGACGACGATAAA; reverse: ATATGTCGACTCAGTGGTGGTGATGGTGATGATGATGTCTAGAGCTATTCAGATCCTC. The resulting amplicon was then inserted into NheI-SalI sites of pCMV-FLRT3*^ECD^*-Fc to allow full substitution and prevent Ig expression by introduction of an upstream stop codon.

**Image acquisition and analysis**

Images from immunofluorescence and actin-staining samples were acquired and analyzed using a confocal microscope Leica SP8 and samples were randomly collected on the basis of receptor and/or mVenus expression. The experimentator was blinded as to the outcome e.g. morpholigical parameters but not regarding group allocation. For actin-dependent structures determination and cell morphology, images were captured using a 100X objective and collecting frame-wise 0.4 µm horizontal z-stacks at a 2X zoom. F-actin density analyses were determined through the quantification of panoramic images taken with a 40X objective and collecting frame-wise 10 µm horizontal z-stacks at a 0.75x zoom. Data for cell and nuclei dimensions were obtained from maximal projection images by performing manual tracing procedures using the polygonal tool from Leica LAS AF Lite software. Filopodia, lamellipodia and blebs were manually identified based on the following morphological criteria extracted from the literature ^4, 5^: Filopodia, rod-like protrusions originating from the cell membrane and filled with cortical f-actin; lamellipodia, sheet-like protrusions which must contain cortical f-actin at its periphery; blebs, round-like protrusions which may or may not contain cortical f-actin at its border. Cell height was determined by summing up all individual sections collected as Z-stacks and Pearson coefficient analyses as well as Phalloidin-rhodamine fluorescent pixel intensity (F-actin content) were calculated using Leica software.

**Miscellaneous procedures**

*Cell cultures and transfections−* HEK293 and HEK293T cells were from ATCC and routinely tested for presence of mycoplasma. Cells were transfected using the polyethyleneimine method (PEI, 25 kDa linear; 1 mg/mL in PBS; Polysciences) using a 1:3 DNA:PEI ratio diluted into serum-reduced medium (Opti-MEM; Gibco) or alternatively DMEM medium. Transfection complexes were added to 60-80 % confluent cells and incubated for 16-48 h before fresh media substitution containing 10 % FBS, 2 mM Glutamax^TM^ (Invitrogen), and 0.1 mg/mL penicillin/10 000 U/mL streptomycin (InVitro). Expi293 cells were transfected using the ExpiFectamine™ 293 transfection kit (ThermoFisher) following manufacturer’s instructions.

*Recombinant ligands production and purification−* Expi293 cells were from ThermoFisher (A14 and routinely tested for the presence of mycoplasma. Media from transfected Expi293 cells were collected 96 h post-transfection and cleared by centrifugation before adding protein-A Sepharose beads (GE healthcare) to immobilize FLRT3*^ECD^*-Fc or Ni^2+^-NTA agarose beads (Qiagen) to immobilize Ten2*^ECD^*-His. Fc- and His-tagged proteins were eluted from beads using 4 M MgCl_2_ or 500 mM imidazole respectively, dialyzed using centrifugal devices (Millipore) and analyzed using SDS-PAGE gels which were stained with Coomassie blue for densitometry quantification purposes.

*Immunoblotting procedures−* Transfected cells were harvested in lysis buffer (containing in mM: 50 Tris pH7.4, 150 NaCl, 5 EDTA and 1 % Triton-X100) complemented with protease inhibitor cocktail (Roche), cleared of cell debris by centrifugation and quantified using the Bradford method. 20 µg of total proteins was loaded on 10 % SDS-polyacrylamide gels and subjected to electrophoresis. Gels were then transferred onto nitrocellulose membranes following manufacturer’s instructions. Membranes were processed using standard procedures and incubated either with anti-FLAG rabbit polyclonal antibody (1: 2 000; Sigma Aldrich, F7425) or anti-GAPDH rabbit polyclonal antibody (1: 2 000; Cell Signaling Technology, 3683S). Membranes were scanned for peroxidase signal using Fusion Pulse photo-documentation system (Vilber Lourmat).

**References**

1. Cruz-Ortega JS, Boucard AA. Actin cytoskeleton remodeling defines a distinct cellular function for adhesion G protein-coupled receptors ADGRL/latrophilins 1, 2 and 3. *Biol Open* 2019; **8**(4).

2. Domene S, Stanescu H, Wallis D, Tinloy B, Pineda DE, Kleta R *et al.* Screening of human LPHN3 for variants with a potential impact on ADHD susceptibility. *Am J Med Genet B Neuropsychiatr Genet* 2011; **156B**(1)**:** 11-18.

3. Silva JP, Lelianova VG, Ermolyuk YS, Vysokov N, Hitchen PG, Berninghausen O *et al.* Latrophilin 1 and its endogenous ligand Lasso/teneurin-2 form a high-affinity transsynaptic receptor pair with signaling capabilities. *Proc Natl Acad Sci U S A* 2011; **108**(29)**:** 12113-12118.

4. Hernandez-Vasquez MN, Adame-Garcia SR, Hamoud N, Chidiac R, Reyes-Cruz G, Gratton JP *et al.* Cell adhesion controlled by adhesion G protein-coupled receptor GPR124/ADGRA2 is mediated by a protein complex comprising intersectins and Elmo-Dock. *J Biol Chem* 2017; **292**(29)**:** 12178-12191.

5. Laser-Azogui A, Diamant-Levi T, Israeli S, Roytman Y, Tsarfaty I. Met-induced membrane blebbing leads to amoeboid cell motility and invasion. *Oncogene* 2014; **33**(14)**:** 1788-1798.

**Supplementary figures and figure legends**

**Supplementary Figure 1| Purification of soluble recombinant Lphn3 ligands, FLRT3 and Teneurin2. (A)** Coomassie-stained SDS-PAGE gel of purified soluble ligands FLRT3*^ECD^*-Fc, Teneurin2*^ECD^*-His and Ig-Fc (negative control), expressed in Expi293 cells. **(B)** Schematic representations of the constructions corresponding to soluble recombinant ligands; indicated domains are the following: Fc IgG, constant fraction of immunoglobulin; FNIII, fibronectin type III; LRRCT, leucine rich repeat C-terminal domain; LRR, leucine-rich repeats; LRRNT, leucine rich repeat N-terminal domain; EGF, epidermal growth factor repeats; Tox, Toxin. Abbreviations: A.M.W, apparent molecular weight; C.M.W, calculated molecular weight.

**Supplementary Figure 2| Cell surface labelling assays of HEK293T heterogeneously expressing Lphn3 receptor variants using FLRT3 extracellular domain recombinant protein.** Cell surface labelling assay displaying indicated receptor-expressing cells visualized using anti-Flag antibody (Lphn3-NTF; red fluorescent signal from secondary antibody coupled to Alexa633) and cell surface-bound FLRT3*^ECD^*-Fc ligand detected with an anti-human IgG antibody (green fluorescent signal from secondary antibody coupled to Alexa488). Note that the signal corresponding to cell surface-bound ligand is strictly restricted to cells expressing Lphn3 receptors, which denotes that naive HEK293T cells do not express detectable amounts of endogenous FLRT3 receptors (such as presumably FLRT isoforms, FGFR or Unc5 for example). Data consist of representative images obtained from at least three separate experiments for which similar results were observed.

**Supplementary Figure 3| Saturation binding curves characterizing the interaction between Lphn3 ADHD-related receptor variants and recombinant ligands FLRT3 or Teneurin-2.** Binding assays conducted with ligands FLRT3*^ECD^*-Fc **(A-E)** or Teneurin2*^ECD^*-His **(F-J)** with cells expressing Lphn3-WT or indicated receptor variants. Affinity parameter, Kd (dotted red line), as well as maximal saturation binding levels, Bmax (dotted green line), were obtained from saturation binding curves adjusted following a non-linear regression calculation. Data are representative binding curves from at least 3 separate experiments (n=3) in which the specific signal was obtained from subtracting the signal detected for mock-transfected cells to total binding observed in Lphn3 receptor-expressing cells.

**Supplementary Figure 4| Functional validation of BRET-based biosensors.** **(A-G)** (*left panels*) Kinetic traces representing BRET^2^ signals elicited by indicated BRET-based biosensors co-expressed with indicated control GPCRs in absence or presence of given GPCR-specific ligands (arrow). Data represent means ± SEM of triplicate in a representative experiment that was repeated at least three times with similar results. **(A-G)** (*right panels*) Inversion**-**BRET biosensors activation profile represented by the iBRET index, in relation to increasing receptor expression levels as a result of increasing DNA concentrations (0-750 ng) and assessing their intrinsic ability to induce functional coupling to G proteins. Biosensors evaluated in each panel: Gαi1 biosensor response to OPRL1 nociceptin-induced activity (*left*) and expression level-dependent constitutive activity (*right*) **(A)**, Gαi2 biosensor response to OPRL1 nociceptin-induced activity (*left*) and expression level-dependent constitutive activity (*right*) **(B)**, Gαi3 biosensor response to OPRL1 nociceptin-induced activity (*left*) and expression level-dependent constitutive activity (*right*) **(C)**,Gαs biosensor response to β2AR isoproterenol-induced activity (*left*) and expression level-dependent constitutive activity (*right*) **(D)**, Gαq biosensor response to AT1 angiotensinII-induced activity (*left*) and absence of expression-level dependent constitutive activity (*right*) **(E)**, Gα12 biosensor response to TPα U46619-induced activity (*left*) and GPR56 expression level-dependent constitutive activity (*right*) **(F)** and Gα13 response to TPα U46619-induced activity (*left*) and GPR56 expression level-dependent constitutive activity (*right*) **(G)**.  Arrows in the left panels indicate addition of indicated compounds or buffer (negative control). OPRL1: opioid-related nociception receptor 1; β2AR: β2-adrenergic receptor; AT1: angiotensin II receptor type 1; TPα: thromboxane A2 receptor α. *P* values describing significance between 0ng DNA (dotted line) and a given DNA concentration: # *P*<0.05, ##*P*<0.01, ### *P* <0.001, #### *P* <0.0001. Data are representative of at least 3 separate experiments (n=3)

**Supplementary Figure 5| BRET^2^ ratio plots for BRET-based biosensors determined in cells expressing increasing amounts of Lphn3 or its ADHD-related variants.** Representative BRET^2^ ratio traces in relation to increasing expression levels of the indicated receptor as a result of increasing DNA concentrations (0-750 ng) and assessing their intrinsic ability to induce functional coupling with: Gαi1 **(A)**, Gαi2 **(B)**, Gαi3 **(C)**, Gαs **(D)**, Gαq **(E)**, Gα12 **(F)** and Gα13 **(G)**. The baseline was set as the BRET^2^ value corresponding to the absence of receptor expression (dotted red line). Note that activation of a given biosensor is represented by a decrease in BRET^2^ values compared to the baseline value. The dotted green line in panel G represents the biosensor BRET^2^ ratio signal value reached by the condition with maximum Lphn3-WT receptor expression (750 ng of plasmid DNA). Graphs are representative results of data obtained from at least 3 separate experiments including four replicates each (n=3). *P* values describing significance between 0ng DNA and a given DNA concentration within the indicated receptor variant group: * *P* <0.05, ** *P* <0.01, *** *P* <0.001, **** *P* <0.0001.

**Supplementary Figure 6| F-actin content analysis of cells expressing Lphn3 ADHD-related variants reveals a disequilibrium in actin dynamics displayed by R465S-expressing cells. (A-F)** Representative images from confocal microscopy analyses of cells expressing mVenus only or mVenus-fused Lphn3 receptors variants (green). Cells were stained with phalloidin-rhodamine to detect f-actin levels (magenta) and with DAPI for nuclei identification (cyan). Scale bar = 50 µm **(**G**)** Quantification of the sum of total phalloidin-rhodamine pixel intensity per mVenus or receptor-mVenus expressing-cells corresponding to total f-actin content**.** F-actin content is higher in mVenus-expressing cells than in Lphn3-expressing cells. Comparing Lphn3 receptor variants to Lphn3-WT reveals a higher f-actin content in R465S-expressing cells. **(H)** Quantification of f-actin density expressed as a ratio between the sum of total phalloidin-rhodamine pixel intensity over respective cell area (pixels/μm^2^) as a corrective factor to panel G giving that Lphn3 expressing cells display a smaller size than control mVenus-expressing cells. Amongst all ADHD-related Lphn3 receptor variants the R465S receptor variant possesses a higher f-actin density then its wild-type counterpart. Data in G and H are represented as the mean values of at least 3 independent experiments (mVenus n=60, WT n=68, A247S n=70, R465S n=71, D615N n=68, T783M n=65). **(I-N)** Scatter distribution diagram displaying the repartition of data obtained in this study illustratively representing the correlation between f-actin density and receptor density into 4 quadrants (numbers indicate data repartition percentage with no statistical value given the illustrative nature of the diagram): Q1= low receptor density - high f-actin density; Q2= high receptor density - high f-actin density; Q3= high receptor density - low f-actin density; Q4= low receptor density - low f-actin density. Note that Lphn3-WT expression does not change the repartition pattern of f-actin density with receptor expression compared to mVenus condition. However, all other receptor variants increase the repartition of high f-actin density within the higher receptor expression quadrant. Notably, R465S almost doubles the repartition of f-actin into the high-density quadrant coinciding with high receptor expression. Also, cells expressing low R465S receptor density immediately led to a relocation of f-actin into the high-density quadrant.

**Supplementary Figure 7| Persistent intercellular contacts between FLRT3 and Lphn3 ADHD-related variants maintain the constitutive activity elicited by the receptors on BRET-based Gα protein biosensors for Gαs, Gαi and Gαq. (A-O)** Plots illustrating the normalized BRET^2^ percentage ratio where the maximum BRET^2^ ratio (100%), obtained after a 24h co-culture period, represents the condition in absence of receptor in sensor-cells but in the presence of empty vector-transfected cells (pCMV) or inducer-cells expressing FLRT3 ligand (FLRT3). **(A-E)** Normalized BRET^2^ ratio plots depicting the activity of the Gαi biosensor within read-out cells expressing Lphn3-WT receptor **(A)** and the indicated ADHD-related variants **(B-E)**. **(F-J)** Normalized BRET^2^ ratio plots depicting the activity of the Gαs biosensor within read-out cells expressing Lphn3-WT receptor **(F)** or the indicated ADHD-related variants **(G-J)**. **(K-O)** Normalized BRET^2^ ratio plots depicting the activity of the Gαq biosensor within read-out cells expressing Lphn3-WT receptor **(K)** or the indicated ADHD-related variants **(L-O)**. Data are represented as mean values obtained from at least 3 independent experiments each containing four replicates. Error bars indicate standard error of means.

**Supplementary Figure 8| Persistent intercellular contacts between Teneurin-4 and Lphn3 variants do not alter the constitutive activity elicited by the receptors on BRET-based Gα protein biosensors for Gα13, Gαi and Gαq. (A-O)** Plots illustrating the normalized BRET^2^ percentage ratio where the maximum BRET^2^ ratio (100%), obtained after a 24h co-culture period, represents the condition in absence of receptor in sensor-cells but in the presence of empty vector-transfected cells (pCMV) or inducer-cells expressing Teneurin-4 ligand (Ten4). **(A-E)** Normalized BRET^2^ ratio plots depicting the activity of the Gα13 biosensor within read-out cells expressing Lphn3-WT receptor **(A)** or the indicated ADHD-related variants **(B-E)**. **(F-J)** Normalized BRET^2^ ratio plots depicting the activity of the Gαi biosensor within read-out cells expressing Lphn3-WT receptor **(F)** or the indicated ADHD-related variants **(G-J)**. **(K-O)** Normalized BRET^2^ ratio plots depicting the activity of the Gαq biosensor within read-out cells expressing Lphn3-WT receptor **(K)** or the indicated ADHD-related variants **(L-O)**. Data are represented as mean values obtained from at least 3 independent experiments each containing four replicates. Error bars indicate standard error of means.
