# Supplementary figures and images for: Convergent selective signaling impairment exposes the pathogenicity of latrophilin-3 missense variants linked to inheritable ADHD susceptibility
Source: Mol Psychiatry. 2022 Apr 7;27(5):2425–38. doi: 10.1038/s41380-022-01537-3 (PMC9135631; doi:10.1038/s41380-022-01537-3)

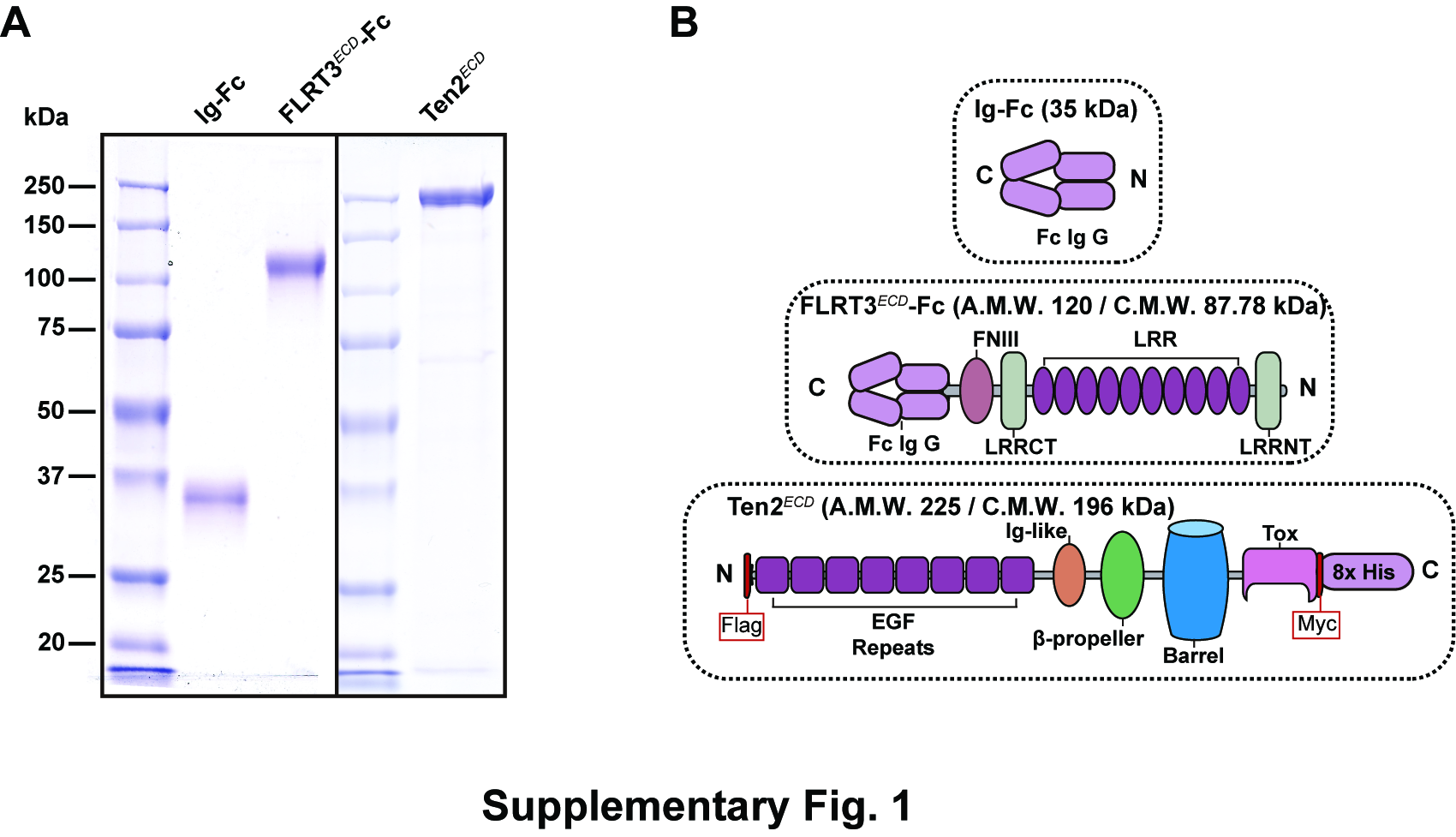

Supplement: Supplementary file 2 — Supplementary Figure 1. Purification of soluble recombinant Lphn3 ligands, FLRT3 and Teneurin2. [file 41380_2022_1537_MOESM2_ESM.tif]

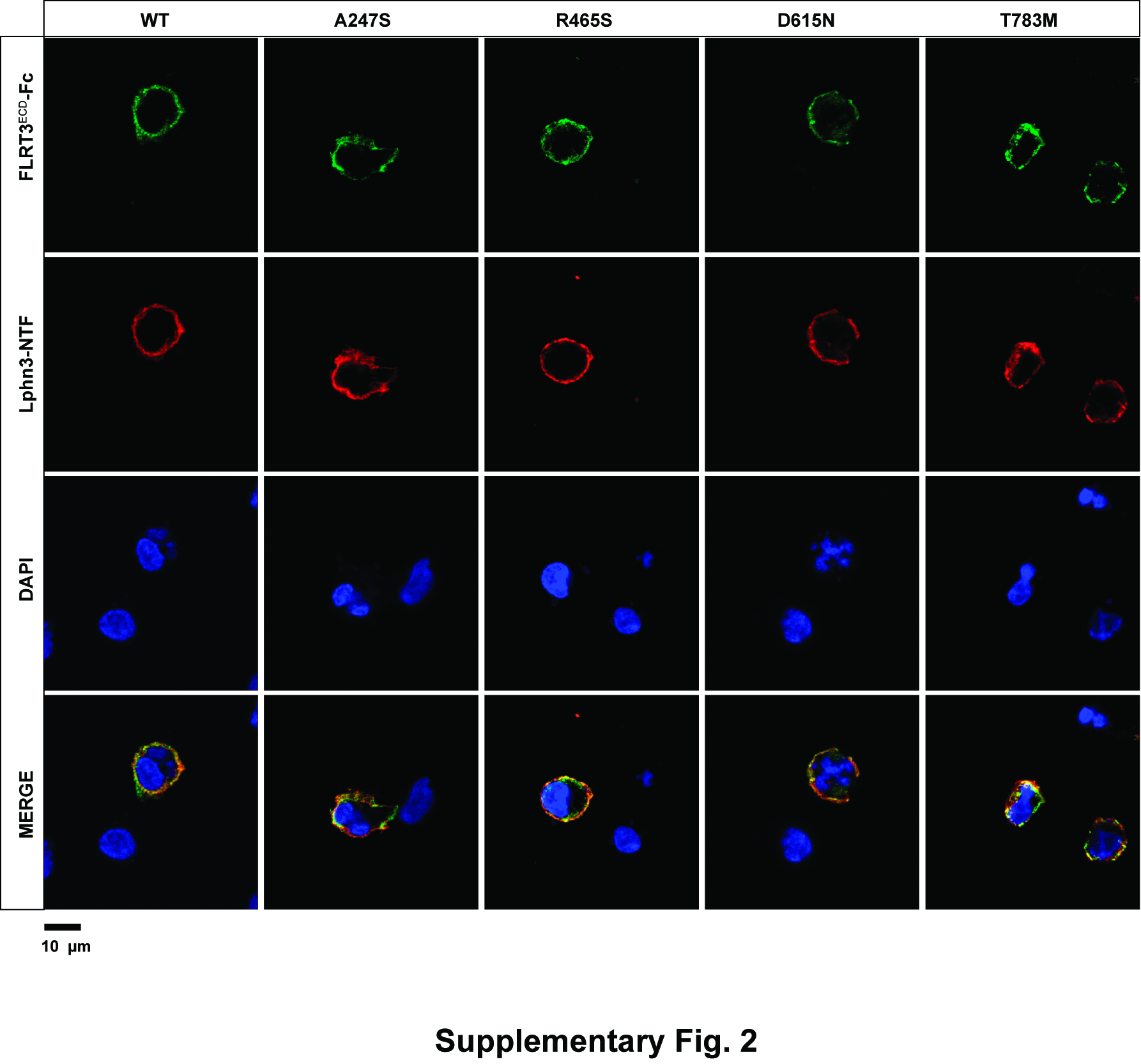

Supplement: Supplementary file 3 — Supplementary Figure 2. Cell surface labelling assays of HEK293T heterogeneously expressing Lphn3 receptor variants using FLRT3 extracellular domain recombinant protein. [file 41380_2022_1537_MOESM3_ESM.tif]

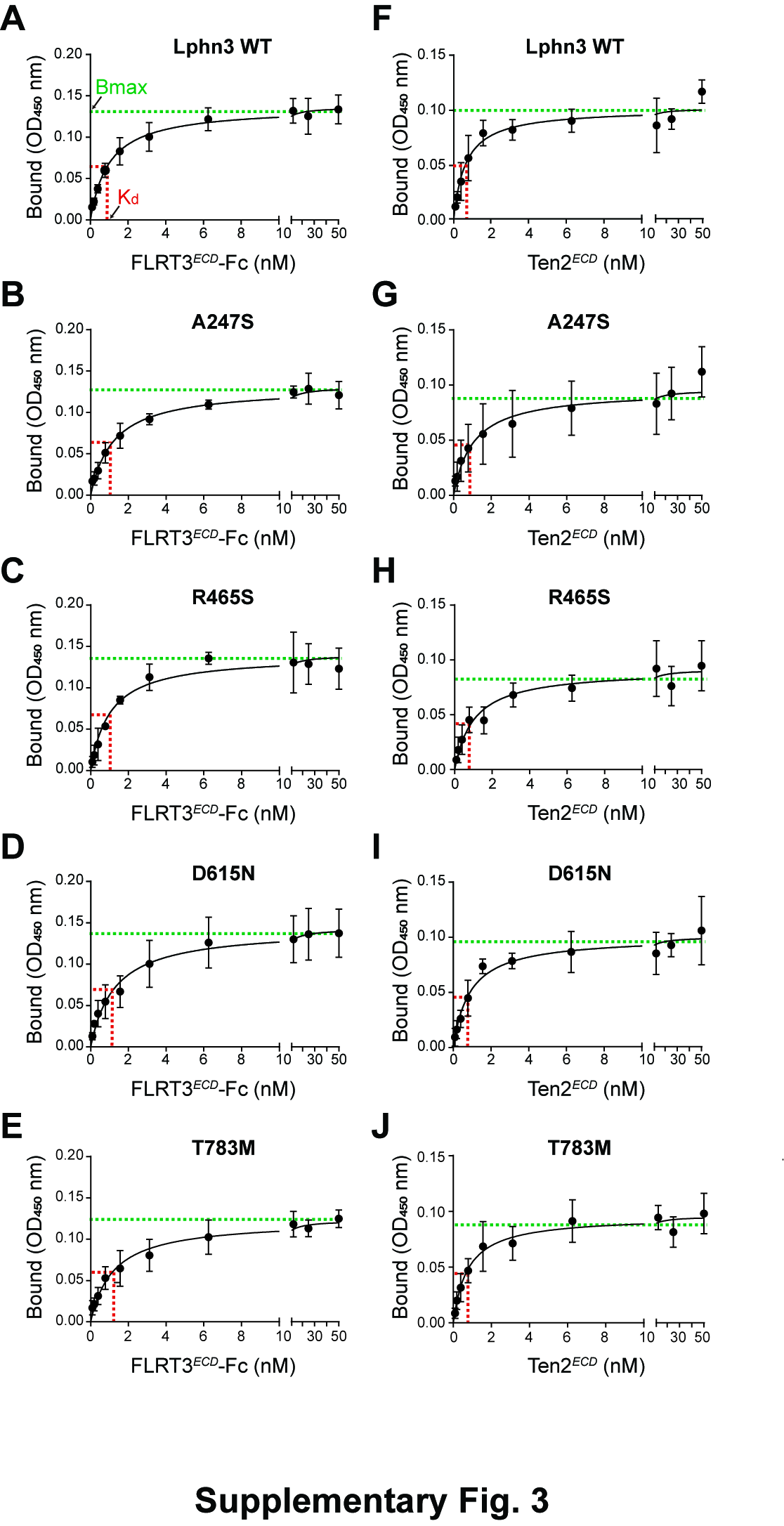

Supplement: Supplementary file 4 — Supplementary Figure 3. Saturation binding curves characterizing the interaction between Lphn3 ADHD-related receptor variants and recombinant ligands FLRT3 or Teneurin-2. [file 41380_2022_1537_MOESM4_ESM.tif]

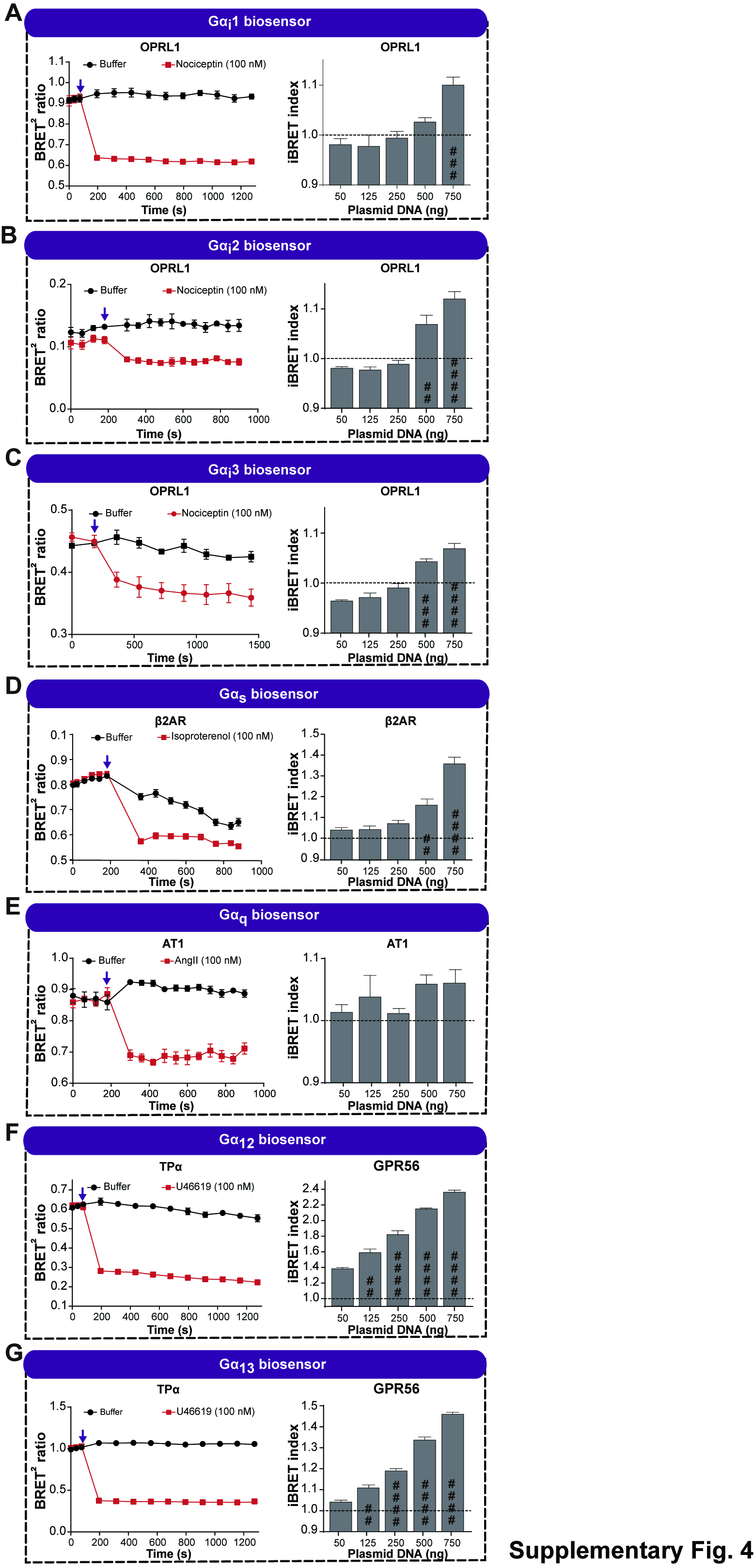

Supplement: Supplementary file 5 — Supplementary Figure 4. Functional validation of BRET-based biosensors. [file 41380_2022_1537_MOESM5_ESM.tif]

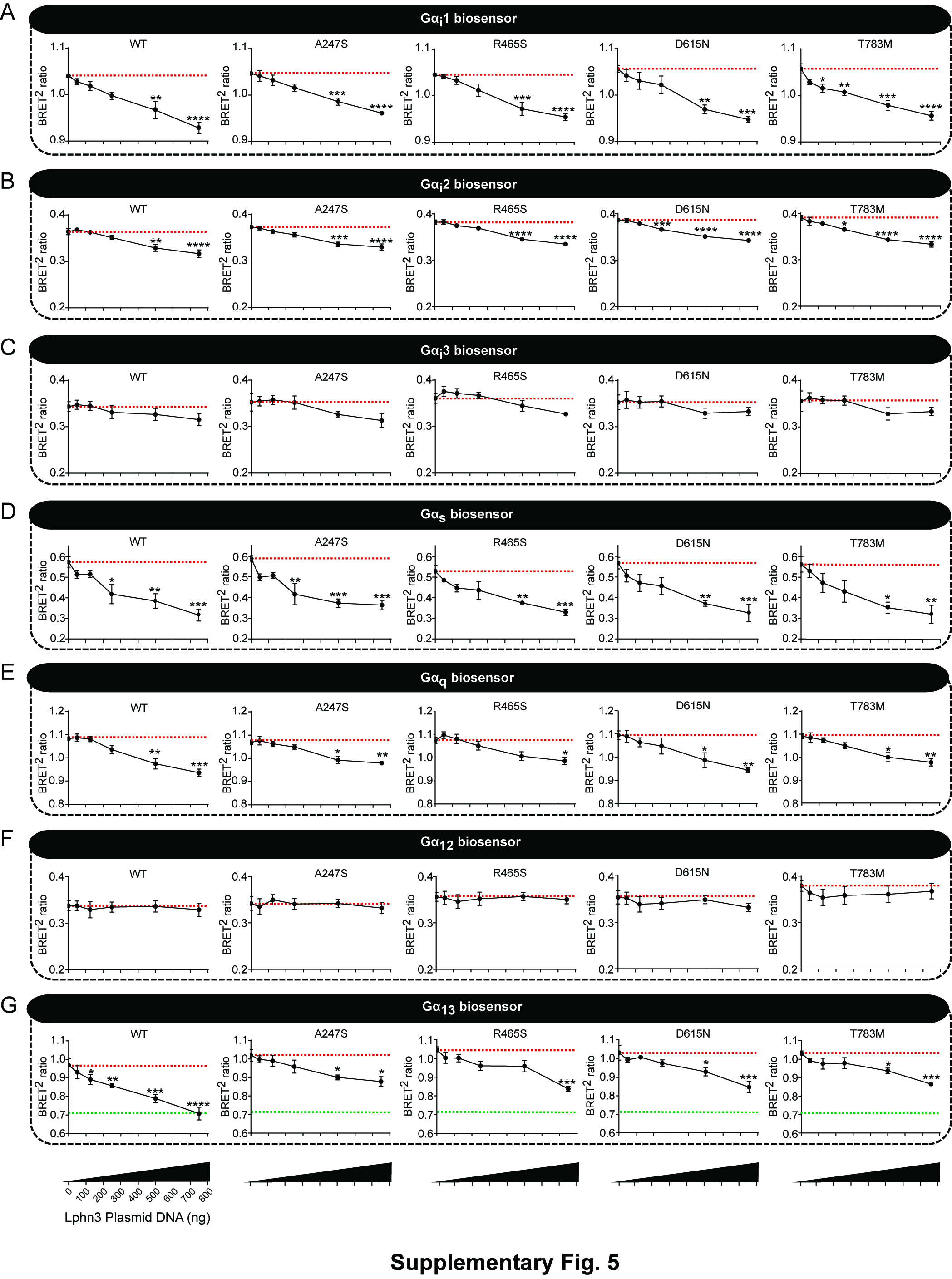

Supplement: Supplementary file 6 — Supplementary Figure 5. BRET2 ratio plots for BRET-based biosensors determined in cells expressing increasing amounts of Lphn3 or its ADHD-related variants. [file 41380_2022_1537_MOESM6_ESM.tif]

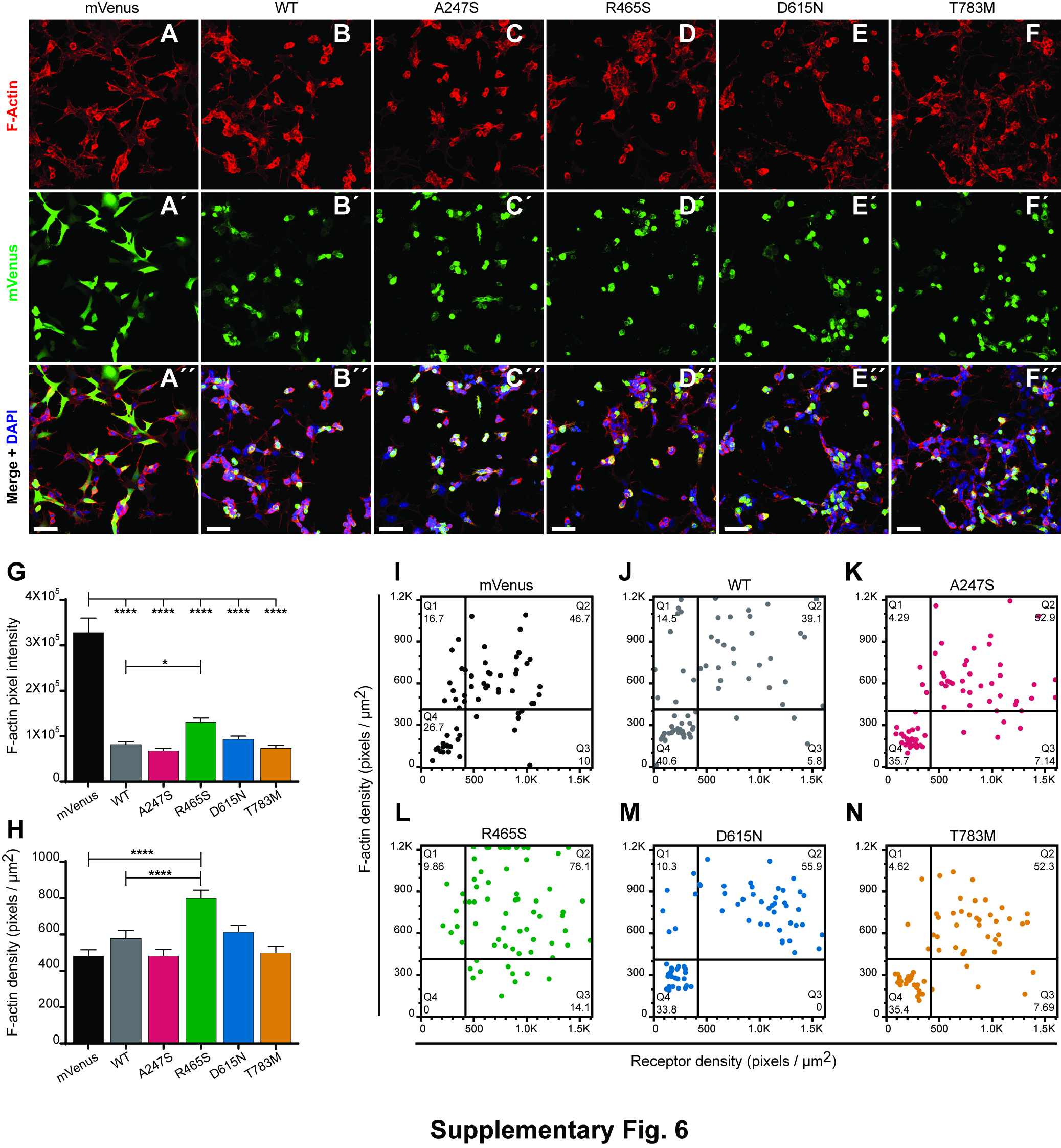

Supplement: Supplementary file 7 — Supplementary Figure 6. F-actin content analysis of cells expressing Lphn3 ADHD-related variants reveals a disequilibrium in actin dynamics displayed by R465S-expressing cells. [file 41380_2022_1537_MOESM7_ESM.tif]

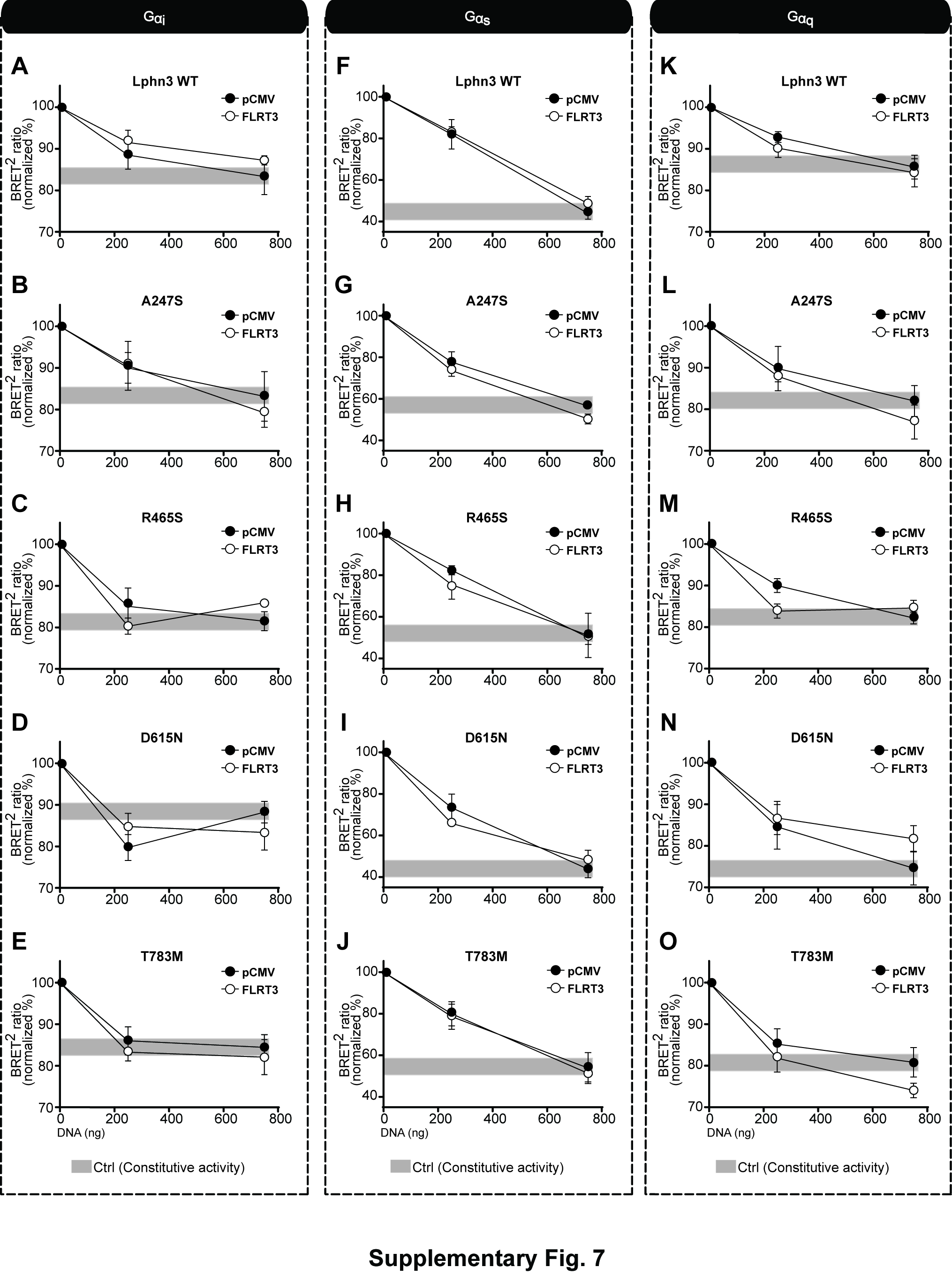

Supplement: Supplementary file 8 — Supplementary Figure 7. Persistent intercellular contacts between FLRT3 and Lphn3 ADHD-related variants maintain the constitutive activity elicited by the receptors on BRET-based Gα protein biosensors [file 41380_2022_1537_MOESM8_ESM.tif]

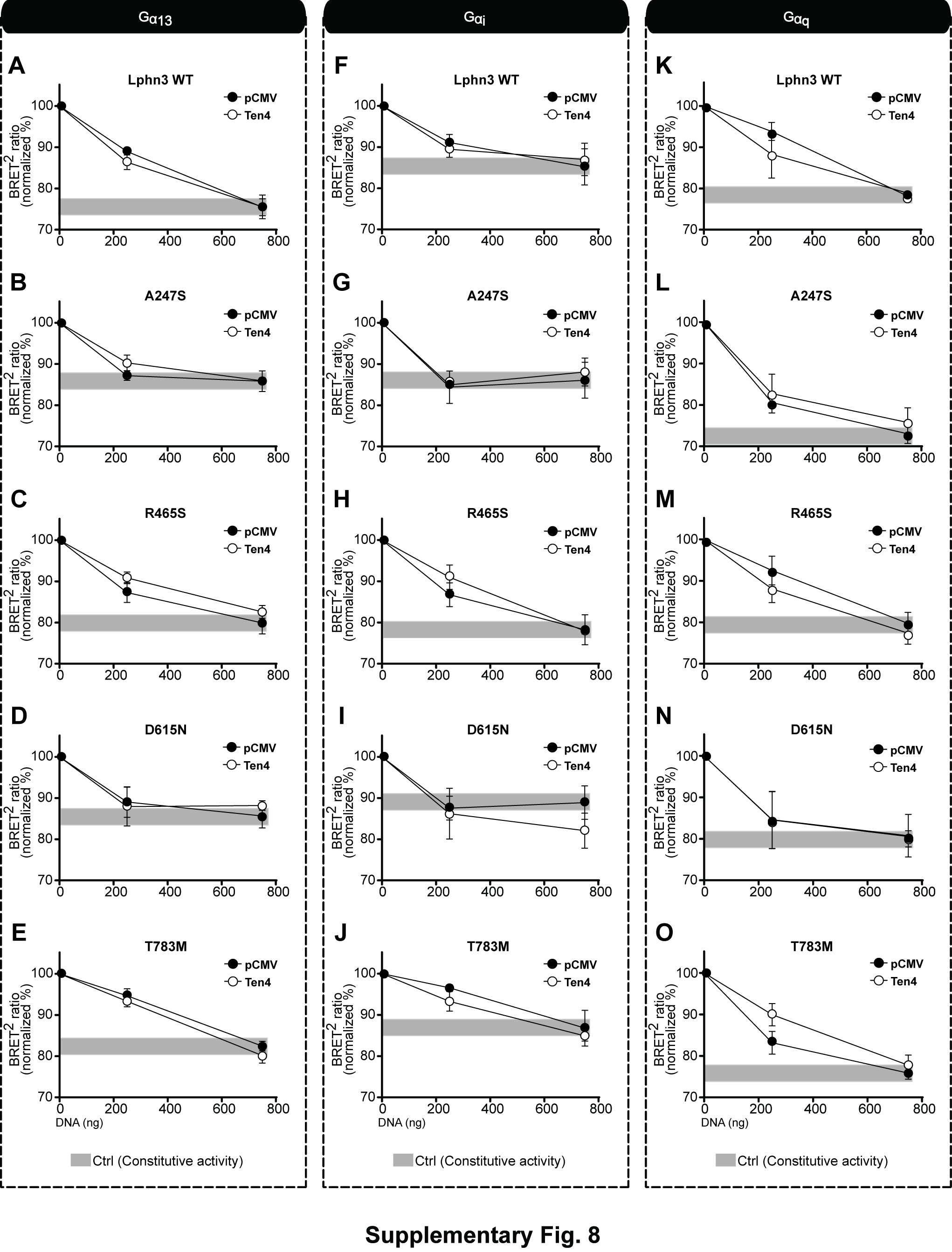

Supplement: Supplementary file 9 — Supplementary Figure 8. Persistent intercellular contacts between Teneurin-4 and Lphn3 variants do not alter the constitutive activity elicited by the receptors on BRET-based Gα protein biosensors [file 41380_2022_1537_MOESM9_ESM.tif]
